# Repetitive transcranial magnetic stimulation for post-stroke non-fluent aphasia: a systematic review and meta-analysis of randomized controlled trials

**DOI:** 10.3389/fneur.2024.1348695

**Published:** 2024-05-01

**Authors:** Jing Cheng, Yijing Jiang, Ting Rao, Yihan Yang, Yanping Liu, Ying Zhan, Shanli Yang

**Affiliations:** ^1^First School of Clinical Medicine, Fujian University of Traditional Chinese Medicine, Fuzhou, China; ^2^Affiliated Rehabilitation Hospital of Fujian University of Traditional Chinese Medicine, Fuzhou, China; ^3^School of Rehabilitation Medicine, Fujian University of Traditional Chinese Medicine, Fuzhou, China

**Keywords:** stroke, non-fluent aphasia, meta-analysis, repetitive transcranial magnetic stimulation, systematic review

## Abstract

**Objective:**

To systematically evaluate the efficacy and safety of repetitive transcranial magnetic stimulation (rTMS) on language function in patients with non-fluent aphasia post-stroke.

**Methods:**

We selected randomized clinical trials (RCT) that involved stroke patients with non-fluent aphasia, whose intervention was rTMS vs. no therapy or other therapy. Two researchers autonomously reviewed the literature based on the specified criteria for inclusion and exclusion and completed the process of data extraction, data verification, and quality evaluation. Meta-analysis was performed using RevMan 5.4^1^ and Stata MP 17^2^, while the assessment of risk of bias was carried out utilizing the Risk of Bias version 2 tool (RoB2)^3^.

**Results:**

The meta-analysis involved 47 RCTs, encompassing 2,190 patients overall. The indexes indicated that rTMS has the potential to decrease the severity of non-fluent aphasia in stroke patients, including improvement of the capability of repetition, naming, and spontaneous language. The determination of BDNF in the serum of patients was also increased. In addition, rTMS reduced the likelihood of depression in stroke patients.

**Conclusion:**

To summarize the relevant studies, rTMS has significant effects on improving the language abilities of stroke patients suffering from non-fluent aphasia, including the abilities of repetition, naming, and spontaneous language.

## Introduction

Stroke remains a primary cause of mortality and morbidity globally (1, 2). Approximately 38% of adult stroke victims are subsequently diagnosed with aphasia (3, 4), which further worsens the prognosis for these patients. The severity of aphasia is useful in predicting the functional autonomy of patients, as well as their short-term and long-term recovery outcomes following a stroke (5, 6). Patients suffering from post-stroke aphasia (PSA) exhibit significantly elevated mortality rates and poorer functional outcomes compared to those without the condition (7). Therefore, rehabilitation therapy after a stroke places a paramount emphasis on the restoration of language function.

Non-fluent aphasia (NFA), frequently observed in individuals recovering from stroke, results from damage to areas encompassing the left inferior frontal gyrus (IFG-L), namely Broca’s area, along with transcortical motor, global, and mixed transcortical aphasia (8, 9). Patients with NFA may exhibit notable impairments in language production, poor sentence repetition, poor verbal agility, and errors in sentence construction (9–11). Traditional speech and language therapies (SLTs) rarely lead to the complete restoration of linguistic functions. Therefore, it is clear that patients with NFA after stroke require more adjunctive and enhancing therapies (12).

Repetitive transcranial magnetic stimulation (rTMS) utilizes magnetic fields to elicit electrical currents within targeted areas of brain. This technique modulates cortical excitability in both the stimulated areas and distant regions by delivering consistent stimuli at extremely short intervals. This process helps restore inter-hemispheric balance and allows for precise control of stimulation parameters (frequency and location), significantly affecting the functional brain network (13). rTMS induces long-lasting neuroplastic changes and facilitates network-related brain reconstruction (14). Theta-burst stimulation (TBS), an advanced form of rTMS, is subdivided into intermittent theta-burst stimulation (iTBS) and continuous theta-burst stimulation (cTBS) (15). Different rTMS stimulation frequencies yield varied effects on cerebral cortex activity: high-frequency stimulation (≥5 Hz) and iTBS enhance local neuronal excitability, whereas low-frequency stimulation (≤1 Hz) and cTBS reduce it (16).

Martin et al. (17) first reported to support language recovery, yet conclusive findings are elusive, and influenced by various factors. An important consideration is whether SLT should be paired with rTMS. Several studies suggested that when employed as a standalone therapy, rTMS holds promise in producing language improvements in PSA (6, 18–20). On the contrary, the suggestion that rTMS could prime the brain for behavioral therapy, implying that it should be integrated with SLT, is met with challenges. Heterogeneity in SLT types and intensities among recent studies used alongside rTMS (21–24) contribute to the complexity of this argument. It is challenging to precisely define the individual contributions of rTMS and SLT and assess their collective impact on PSA rehabilitation that rTMS could offer a unique, complementary approach to treating aphasia. Current research into PSA rehabilitation has used rTMS to modulate interhemispheric interaction.

Numerous randomized controlled trials (RCTs) (25) suggest that rTMS may aid in the reconstruction and recovery of language abilities in individuals with PSA. Six systematic reviews (25–30) evaluated the impact of rTMS on PSA, with most reaching inconsistent conclusions or having loose exclusion criteria; the types of aphasia in patients were also ambiguously defined. Georgiou et al. (31) utilized the AMSTAR 2 tool to evaluate the quality of systematic reviews of RCTs focusing on the effectiveness of rTMS in aphasia rehabilitation following stroke before July 2017 and found that the quality of these studies was generally low. Another meta-analysis (32) identified the types of NFA in stroke patients, but the included literature was outdated. Consequently, we conducted a systematic review to furnish evidence-based information regarding the application of rTMS in treating NFA following a stroke. This involved analyzing a large number of studies and more relevant outcome indices, to identify new research directions.

## Methods

This systematic review adheres to the guidelines set forth by the Preferred Reporting Items for Systematic Reviews and Meta-Analyses (PRISMA) guidelines (33). And the study protocol has been officially registered in PROSPERO (ID CRD42023434714).

Our PICO question was: in stroke patients with NFA, does rTMS, as compared to the absence of therapy or alternative treatments, reduce the severity of aphasia in patients, including naming, spontaneous language, and repetition abilities?

### Search strategy

We searched nine commonly used electronic databases: PubMed, Cochrane Library, Embase, Web of Science, SinoMed, OVID, the China National Knowledge Infrastructure (CNKI), China Science and Technology Journal Database (VIP), and Wanfang Data for RCTs of rTMS for stroke patients with NFA. Furthermore, relevant systematic evaluations and reference lists of included studies were searched manually to ensure the comprehensiveness of included studies. Keywords were determined after preretrieval: repetitive transcranial magnetic stimulation, rTMS, TBS, stroke, cerebrovascular accident, aphasia, non-fluent aphasia, and post-stroke aphasia. The final literature search was conducted on January 8, 2024, using neither language nor publication date restrictions.

### Inclusion and exclusion criteria

Inclusion criteria (1) study design: RCTs; (2) study population: individuals with NFA following a clinical diagnosis of stroke; (3) interventions: In addition to the interventions applied to the control group, the experimental group underwent rTMS. Alternatively, the experimental group received rTMS, whereas the control group received sham-rTMS. For studies encompassing more than two groups, the groups fulfilling the inclusion criteria were also included; (4) the outcome indicators ought to incorporate at least one metric of aphasia assessment, such as Western Aphasia Battery (WAB), Aphasia Battery of Chinese (ABC), China Rehabilitation Research Center Standard Aphasia Examination (CRRCAE), and Boston Naming Test (BNT).

Exclusion criteria: (1) duplicate studies or data cannot be extracted; (2) case studies, animal experimental studies, or reviews; (3) the unavailability of full text even after contacting the author via email.

### Study selection and data extraction

Endnote X9 was utilized for document organization and deduplication. Two reviewers independently conducted literature screening, data extraction, and cross-verification according to predetermined inclusion and exclusion criteria. Any discrepancies between the reviewers were resolved by achieving a consensus with an unbiased third-party researcher. The process of extracting data entailed gathering details regarding the title, first author, publication year, the number of patients, diagnostic criteria, intervention and control protocols, rTMS parameters (e.g., stimulation site, frequency, intensity, and duration), outcome measures, any reported adverse events and follow-up duration.

#### Risk-of-bias

Risk of bias assessment for the studies included was carried out utilizing the RoB2 tool (34). Two researchers evaluated the risk of bias independently, with any discrepancies being resolved by a third researcher.

### Statistical analysis

Meta-analysis was conducted utilizing Revman 5.4 software. The mean differences (MD) along with the 95% confidence interval (CI) were employed for statistical analysis, and the standardized mean difference (SMD) was used when using different measurement methods or units. Statistically significant differences were indicated by *p* < 0.05, and the magnitude of heterogeneity was quantified using *I*^2^. *I*^2^ shows the proportion of heterogeneity in the total variation of effect size based on the Student–Newman–Keuls test, ranging from 0 to 100%. *I*^2^ ≤ 50% was deemed as an indication of low heterogeneity, employing the fixed-effects model for Meta-analysis; *I*^2^ > 50% was considered a clear indication of significant heterogeneity, utilizing the random-effects model.

## Results

### Search results

The initial search returned 1,244 articles. Following the removal of duplicate articles, 607 studies were left, and 59 studies remained upon reviewing the titles and abstracts. Finally, 47 studies were included after a rigorous evaluation of the full-text articles (Supplementary Figure S1).

### Characteristics of included studies

Out of the 48 studies (4, 23, 35–80), 37 studies (35–37, 39–49, 51, 52, 54–66, 68, 69, 72–74, 76, 78, 79) had reported diagnostic criteria for stroke and 35 (4, 35–39, 41–53, 55, 57–60, 62–65, 67–70, 74, 76, 77) for aphasia. In 43 studies (4, 23, 35–52, 54–65, 67–76, 78, 80), the age of the patient was reported as mean ± standard deviation, while two studies (53, 66) reported specific age information for each patient, and the age range was reported in two studies (77, 79). Except for two studies (50, 59) that failed to disclose the post-stroke time, the mean course of disease for patients in the other 44 studies (4, 23, 35–49, 51, 52, 54–58, 60–65, 67–80) ranged from 6.9 days to 4.46 years, and two studies (53, 66) reported specific course of disease for each patient. Of the total studies, 39 studies (4, 35–43, 45–48, 51–55, 58–70, 72–74, 77–80) specifically reported that the patients were right-handed, whereas the remaining 9 studies (23, 44, 49, 50, 56, 57, 71, 75, 76) failed to furnish this information. Duration of the intervention in 45 studies (4, 23, 35, 36, 38–40, 42–46, 48–80) spanned from 2 to 4 weeks, except for 2 studies (37, 41) with an intervention duration of 30 days and one (47) with 8 weeks. Regarding the content of the intervention, five studies (49, 50, 59, 71, 80) used rTMS or sTMS alone, and the remaining studies also had aphasia treatment components including SLT, acupuncture, and electroacupuncture (EA). Patients were followed up after treatment in 15 studies (4, 39, 40, 53, 57, 66, 67, 70–74, 77, 78, 80), ranging from 30 days to 12 months.

Adverse events were mentioned in 10 studies (4, 40, 42, 45, 51, 57, 60, 61, 73, 77). Six studies (4, 40, 42, 45, 60, 73) reported headache; three (40, 45, 51) reported dizziness; adverse effects of epilepsy seizures were seen in two studies (61, 77). One study (57) documented the occurrence of adverse events such as disorientation, injuries resulting from falls, and aspiration caused by dysphagia among both control and experimental participants, the ratio of aspiration was the highest in the experimental group (3 cases), and the control group (11 cases), which revealed that the combination of rTMS and speech training could reduce the complications and improve the therapeutic efficiency.

Regarding the NFA type, 16 studies (23, 37, 41, 42, 44, 45, 47–49, 54, 56, 57, 61, 74, 78, 79) included patients with Broca aphasia. Four studies (36, 53, 63, 64) included patients with global aphasia, and 5 studies (39, 43, 72, 73, 76) reported on the number of patients with different types of aphasia. The aphasia types of patients in the three studies (43, 72, 73) were global, Broca’s, and transcortical motor aphasia, and Wang’s (73) study included patients with mixed transcortical aphasia. In addition to the intervention received by the control group, the experimental group in one study (48) received high-frequency TMS (HF-rTMS) on the IFG-L. One study (67) performed bilateral cerebral pulse stimulation, with HF-rTMS (> 1 Hz) stimulating the left cerebral Broca area and low-frequency TMS (≤1 Hz) (LF-rTMS) stimulating the right Broca area; three studies (38, 46, 56) stimulated the right inferior frontal gyrus (IFG-R) with 0.5 Hz. Of the 40 studies with a frequency of 1 Hz, except for one study (53) in which the stimulation site was the right superior temporal gyrus, the stimulation site was the IFG-L. Three of these studies (32, 66, 80) further stimulated Brodmann 45 (pars triangularis) at the site of the IFG-R partition. Three studies (43, 58, 77) proceeded theta burst stimulation (TBS) on patients, one (77) conducted passive cTBS on the cerebellum in patients of the experimental group, and the other two (43, 58) conducted iTBS on the IFG-L. The critical characteristics of the included studies have been summarized in Table 1.

**Table 1:** Basic characteristics of the included studies.

Studies and year	Diagnostic criteria		Total patients (C/E)	Age (years)	Course of disease	Hand-edness	Intervention		rTMS parameters			Duration	Outcome indicators	Follow up (after treatment)
	Stroke	Aphasia					C	E	FRQ (Hz)	Intensity	Stimulation sites			
Cao HY, 2023	CT/MRI	①	90 (30/30)	C:56.97 ± 14.25 E:57.80 ± 11.69	C:49.43 ± 18.14 D E:54.73 ± 17.40 D	R	SLT + Eye tracking training	SLT + Eye tracking training+rTMS	1	80–120% of the MT, 1000 pulses	IFG-R	5d/w × 4w	①	NR
Jiang XC, 2023	CT/MRI	③	50 (25/25)	C:63.57 ± 9.69 E:58.86 ± 12.81	35.04 ± 30.65 D 39.95 ± 28.84 D	R	SLT + S-rTMS	SLT + iTBS	50	80% of the rMT, 600 pulses	IFG-L	6d/w × 4w	①③	NR
Zhu HM, 2023	CT/MRI	①	60 (30/30)	C:58.0 ± 11.8 E:59.7 ± 13.6	C:42.5 D E:34.5 D	R	SLT	SLT + rTMS	1	80% of the rMT, 1,000 pulses	IFG-R	6d/w × 3w	①	NR
Liu SJ, 2023	CT/MRI	②	92 (46/46)	C:50.29 ± 9.768 E:53.41 ± 10.23	C:56.98 ± 31.56 D E:53.26 ± 29.98 D	R	SLT + drug treatment+ S-rTMS	SLT + drug treatment+rTMS	0.5	80% of the rMT	IFG-R	5d/w × 4w	②⑤⑨⑪	NR
Liu SL, 2022	CT/MRI	②⑤	70 (35/35)	C: 63.46 ± 9.57 E: 60.94 ± 7.80	C: 60.06 ± 22.56 D E: 56.89 ± 24.80 D	R	SLT + acupuncture treatment	SLT + acupuncture treatment+ rTMS	1	The sequence pulse is 30 times	IFG-R	20 min/d × 5d/w × 8w	①⑤	NR
Xu DM, 2022	CT/MRI	②	60 (30/30)	C:63.5 ± 2.4 E:63.6 ± 2.5	C:5.1 ± 1.3 M E:5.2 ± 1.4 M	NR	SLT	SLT + rTMS	1	80% of the MT	IFG-R	5d/w × 4w	②⑦	3 M
Zheng Y, 2022	CT/MRI	②	45 (15/15)	C:53.27 ± 14.83 E:50.53 ± 13.85	C:50.20 ± 18.52 D E:52.60 ± 18.06 D	R	SLT + attention function training	SLT + attention function training+rTMS	1	80–70% of the MT, 1000 pulses	BA45	20 min/d × 5d/w × 4w	②⑦⑧	NR
Zhou HY, 2021	CT/MRI	NR	106 (53/53)	C:59.87 ± 7.64 E:61.25 ± 8.41	C:8.91 ± 2.36 W E:9.35 ± 3.27 W	R	SLT	SLT + rTMS	1	90% of the MT	IFG-R	5d/w × 4w	①⑧⑪	NR
Wang GX, 2021	CT/MRI	NR	26 (14/12)	C:52.79 ± 12.80 E:52.42 ± 10.56	C/E:3–6 M	R	SLT	SLT + rTMS	1	90% of the MT, 1200 pulses in total	IFG-R	20 min/d × 5d/w × 2w	①⑤	NR
Li WT, 2021	CT/MRI	①	120 (60/60)	C:50.88 ± 6.09 E:51.02 ± 5.78	C:8.68 ± 2.16 D E:8.57 ± 1.38 D	NR	SLT+ acupuncture	SLT + rTMS+ acupuncture	1	80% of the MT, 50 pulses per sequence, 10 sequences per day	IFG-R	20 min/d × 6d/w × 4w	①	NR
Zhang DH, 2021	CT/MRI	①	30 (10/10)	C:50 ± 08 E:50 ± 13	C:4.4 ± 1.2 M E:3.9 ± 1.0 M	R	SLT	SLT + rTMS	5	80% of the AMT,	IFG-L	5d/w × 2w	PACA	NR
Zhu HM, 2021	CT/MRI	①	30 (10/10)	C:60.4 ± 8.3 E:61.6 ± 14.7	C:228.5 D E:246.5 D	R	SLT + MNTS	SLT + MNTS+rTMS	1	80% of the MT, 1000 pulses	IFG-R	6d/w × 3w	①⑤	NR
Zhu HM, 2020	CT/MRI	①	50 (16/18)	C:60.75 ± 9.00 E:57.89 ± 14.15	C:52.00 D E:69.00 D	R	SLT + MNS	SLT + MNS + rTMS	1	80% of the MT, 1000 pulses	IFG-R	2times/d × 6d/w × 3w	①⑤	NR
Qiu LF, 2020	CT/MRI	①	60 (20/20)	C:52.25 ± 15.00 E:55.00 ± 10.72	C:1.56 ± 1.63 M E:2.12 ± 1.72 M	R	SLT + Schuell stimulation	SLT + Schuell stimulation+rTMS	1	80% of the MT, 1200 pulses per day	IFG-R	5d/w × 4w	①③⑪	NR
Qu YZ, 2020	CT/MRI	①	40 (20/20)	C:67.80 ± 7.32 E:68.60 ± 7.78	C:25.80 ± 11.77 D E:26.50 ± 12.51 D	R	SLT	SLT + rTMS	1	100% of the rMT, 1,200 pulses	IFG-R	5d/w × 2w	①⑨	NR
Qin Q, 2020	NR	①	76 (38/38)	C:57.84 ± 12.49 E:57.94 ± 11.39	NR	NR	HBO	HBO + rTMS	1	80% of the rMT	IFG-R	20 min/d × 5d/w × 4w	①⑦	NR
Chen Y, 2020	CT/MRI	③	30 (15/15)	C:61. 9 ± 10. 7 E:56. 6 ± 15. 1	C:10.7 ± 4.4 W E:9.5 ± 3.3 W	R	SLT + drug treatment+ S-rTMS	SLT + drug treatment+rTMS	1	100% of the rMT	IFG-R	20 min/d × 5d/w × 2w	③⑫	30D
Pan LS, 2019	CT/MRI	②	44 (22/22)	C:64.41 ± 11.53 E:68.85 ± 8.97	C:45.00 ± 21.69 D E:53.65 ± 24.92 D	R	RT + EA	RT + EA + rTMS	5	80% of the MT	IFG-L	12 min/d × 5d/w × 4w	②⑦⑩	NR
Qiao Y, 2019	CT/MRI	②	44 (22/20)	C: 58.64 ± 9.99 E: 60.70 ± 9.74	C: 57.32 ± 16.54 D E: 53.50 ± 19.76 D	NR	RT + EA	RT + EA + rTMS	1	20 sequences, and stimulation intensity ranging from 30 to 50%	IFG-R	20 min/d × 5d/w × 4w	②⑦⑩	NR
Zhang Y, 2019	CT/MRI	①	48 (16/16)	C: 60.7 ± 8.7 E: 60.6 ± 9.1	NR	R	RT + HBO	RT + HBO + rTMS	1	120 sequences,960 pulses	IFG-R	20 min/d × 5d/w × 4w	①⑦	NR
Wang JR, 2018	CT/MRI	②	35 (18/17)	C:55.00 ± 11.35 E:47.07 ± 12.52	C:40.87 ± 21.86 D E:39.40 ± 24.05 D	R	SLT + drug treatment	SLT + drug treatment+rTMS	1	80% of the MT	IFG-R	20 min/d × 5d/w × 4w	①	NR
Ren CL, 2018	NR	①	14 (6/6)	Specific age information for each patient	Specific course of disease for each patient	R	SLT + S-rTMS	SLT + rTMS	1	80% of the MT	STG-R	20 min/d × 5d/w × 3w	①	3 and 6 M
Li ZH, 2018	CT/MRI	①	30 (13/13)	C:68.3 ± 5.8 E:65.3 ± 5.6	C:51.0 ± 9.6 D E:47.5 ± 7.4 D	R	SLT + S-rTMS	SLT + rTMS	1	80% of the MT	IFG-R	20 min/d × 5d/w × 3w	① + QEEG	NR
Chang L, 2018	MRI	①	126 (63/63)	C:66.4 ± 15.8 E:67.3 ± 19.9	C:7.3 ± 3.5 D E:6.9 ± 3.1 D	R	SLT	SLT + rTMS	1	80% of the MT	IFG-R	ST 30 min/d × 15d rTMS 20 min/d × 15d	①	NR
Wu G, 2017	CT/MRI	NR	180 (90/90)	56.91 ± 9.70	C/E:3.47 ± 1.16 D	NR	SLT	SLT + rTMS	0.5	80% of the MT	IFG-R	5d/w × 4w	②	NR
Guo CH, 2016	CT/MRI	①	60 (20/20)	C:64.4 ± 8.5 E:62.1 ± 10.6	C:30.6 ± 9.4 D E:33.1 ± 8.6 D	R	SLT	SLT + rTMS	1	70% of the MT	IFG-R	30 min × 6d/w4w	①	NR
Fan YN, 2016	CT/MRI	①	116 (58/58)	C:65.4 ± 15.9 E:64.4 ± 14.5	C:7.2 ± 3.1 D E:6.9 ± 3.3 D	R	ST + drug treatment	ST + drug treatment+rTMS	1	80% of the MT	IFG-R	20 min/d × 30d	①	NR
Shen Y, 2016	NR	①	40 (20/20)	C: 57.5 ± 11.9 E:60.2 ± 10.5	C:45.1 ± 18.8 D E:50.7 ± 16.3 D	R	drug treatment +ST	drug treatment +ST + rTMS	0.5	90% of the MT, 384 pulses	IFG-R	5d/w × 3w	①⑪	NR
ShanYD,2012	CT/MRI	NR	28 (14/14)	C:67.3 ± 10.9 E:69.7 ± 12.8	C:10.3 ± 9.1 W E:11.6 ± 7.5 W	R	SLT	SLT + rTMS	1	100% maximum intensity	IFG-R	20 min/d × 10d	③	90D
Chen F, 2012	CT/MRI	NR	24 (12/12)	C:65.5 ± 2.5 E:66.5 ± 1.8	C/E: < 7 D	R	SLT	SLT + rTMS	1	80% of the MT	IFG-R	20 min/d × 10d	②	2 W, 2 and 6 M
Chen F, 2011	CT/MRI	NR	15 (7/8)	C:66.5 E:65.7	C/E: <7 D	R	SLT	SLT + rTMS	1	80% MEP	IFG-R	10d	②	NR
Bing-Fong Lin, 2023	MRI	NR	33 (17/16)	C:62.24 ± 14.42 E:54.06 ± 12.12	C:12.18 ± 12.63 M E:9.00 ± 7.30 M	R	SLT + S-rTMS	SLT + rTMS	1	90% of the rMT, 900 pulses	IFG-R	20 min × 5d/w × 2w	④	NR
Trevor A. Low, 2023	NR	①	20 (10/10)	C:63.8 ± 5.6 E:61.5 ± 12.2	C:2.4 Y E:3.2 Y	R	M-MAT+ S-rTMS	M-MAT+rTMS	1	100% of the rMT,1,200 pulses	IFG-R	20 min/d × 5d/w × 2w	⑥	3 M
Yaşa İ, C, 2023	MRI	T-RAT	40 (10/10)	C:60.00 ± 5.05 E:59.70 ± 5.31	C:10.4 M E:10.6 M	R	SLT	SLT + rTMS	1	110% of the MT, 1500 pulses	IFG-R	20 min/d × 5d/w × 3w	ADD+ T-PNT	1 M
Guangtao Bai, 2022	CT/MRI	①	60 (30/30)	C:59.91 ± 8.58 E:63.47 ± 7.81	C:3.75 ± 1.67 M E:3.27 ± 1.50 M	R	CST + S-rTMS	CST + rTMS	1	80% of the MT	IFG-R	20 min/d × 5d/w × 4w	①	NR
Kai Zheng, 2022	NR	①	40 (20/20)	40–80	C/E:>6 M	R	SLT + S-rTMS	SLT + rTMS	5	80% of the ATM	Crus I of the right lateral cerebellum	5d/w × 2w	①⑤⑥	12 W
Bing-Fong Lin, 2022	MRI	④	33 (16/17)	C:62.94 ± 14.59 E:54.71 ± 12.03	C:12.63 ± 12.9 M E:9.41 ± 7.27 M	R	SLT + S-rTMS	SLT + rTMS	1	90% of the rMT, 900 pulses	IFG-R	15 min/d × 5d/w × 2w	④	NR
Eun-Ho Yu, 2021	MRI	①	20 (10/10)	C:52.90 ± 10.90 E:59.40 ± 12.18	C:4.63 ± 3.00 M E:5.17 ± 3.30 M	NR	SLT + IBA	SLT + NBA	1	90% of the rMT, 1,200 pulses	IFG-R	20 min × 5d/w × 2w	①	NR
LA Lopez-Romero, 2019	NR	⑥	82 (41/41)	C:65.6 ± 13.4 E:61.9 ± 13.9	C:12.8 M E:9.21 M	R	S-rTMS	rTMS	1	80% of the MT, 1200 pulses	IFG-R	20 min × 5d/w × 2w	⑥	30D
Mohammad Haghighi, 2018	NR	NR	12 (6/6)	C: 60.5 ± 11.85 E:61.67 ± 7.06	C/E:4–8 W	NR	SLT + S-rTMS	SLT + rTMS	1	100% of the rMT	IFG-R	30 min × 5d/w × 2w	①	NR
Tae Hee Yoon, 2015	NR	NR	20 (10/10)	C:61.13 ± 8.72 E:60.46 ± 9.63	C:5.20 ± 2.67 M E:6.80 ± 2.39 M	NR	SLT	SLT + rTMS	1	90% of the rMT, 1,200 pulses	IFG-R	20 min × 5d/w × 4w	①	NR
Chih-Pin Wang, 2014	MRI	NR	45 (15/15)	C:60.4 ± 11.9 E:61.3 ± 13.2	C:16.1 ± 7.3 M E:16.8 ± 6.4 M	R	Naming task+ S-rTMS	Namingtask+ rTMS	1	90% of the rMT, 1,200 pulses	IFG-R	20 min × 5d/w × 2w	④	3 M
Po-Yi Tsai, 2014	MRI	NR	56 (23/33)	C:62.8 ± 14.5 E:62.3 ± 12.1	C:18.3 ± 8.2 M E:17.8 ± 7.2 M	R	SLT + S-rTMS	SLT + rTMS	1	90% of the rMT, 1,200 pulses	IFG-R	10 min × 5d/w × 2w	④	3 M
Eman M. Khedr, 2014	NR	ASRS	30 (10/20)	C:57.4 ± 9.6 E:61.0 ± 9.8	C:4.0 ± 2.6 W E:5.8 ± 4.08 W	R	SLT + S-rTMS	SLT + rTMS	1 / 20	110% of the rMT, 1,000 pulses	right and left Broca	5d/w × 2w	HSS	1 and 2 M
Caroline H.S, 2013	MRI	NR	12 (6/6)	specific age information for each patient	specific course of disease for each patient	R	S-rTMS	rTMS	1	90% of the rMT, 1,200 pulses	BA 45	20 min × 5d/w × 2w	①⑥	2, 8, and 12 M
Jared Medina, 2012	NR	⑤	10 (5/5)	C:62.6 ± 10.1 E:60.6 ± 7.1	C: 58.6 ± 34.8 D E: 49.8 ± 29.6 D	NR	S-rTMS	rTMS	1	90% of the rMT, 1,200 pulses	IFG-R	5d/w × 2w	①	2 M
Barwood CH, 2011	NR	NR	12 (6/6)	C:60.8 ± 5.98 E:67 ± 13.22	C: 3.49 ± 1.27 Y E: 4.46 ± 1.53 Y	R	S-rTMS	rTMS	1	1,200 pulses	BA45	20 min × 10d	⑤⑥	2 M

C, control group; E, experiment group; FRQ, frequency; NR, not reported; L, left; R, right; D/d, days; W/w, weeks; M, month/months; Intensity: MT, motor threshold; rMT, resting motor threshold; AMT, active motor threshold; Intervention: SLT, speech-language therapy; S-rTMS: sham repetitive transcranial magnetic stimulation; CST, conventional speech therapy; HBO, hyperbaric oxygen; M-MAT, Multi-modality aphasia therapy; EA, electroacupuncture; RT, routine treatment; Stimulation sites: IFG-R, right inferior frontal gyrus; IFG-L, left inferior frontal gyrus; PSTG-R, right posterior superior temporal gyrus; BA45, Brodmann 45; IBA, involvement of Broca’s area; NBA, non-IBA; Outcome indicators: ①WAB, Western Aphasia Battery; ②ABC, Aphasia Battery of Chinese; ③CRRCAE, China Rehabilitation Re-search Center Standard Aphasia Examination; ④CCAT, Concise Chinese Aphasia Test; ⑤BDAE, Boston Diagnostic Aphasia Examination; ⑥BNT, Boston Naming Test; ⑦MBI, Modified Barthel Index; ⑧MMSE, Mini-mental State Examination; ⑨NIHSS, National Institutes of Health Stroke Scale; ⑩HAMD, Hamilton Rating Scale for Depression; ⑪CADL, Comprehensive Activities of Daily Living; ⑫FIM, Functional Independence Measure.

### Methodological quality

The outcomes of the risk-of-bias assessments conducted for each included study are shown in Supplementary Figures S2A,B.

Potential publication bias across the included studies was evaluated based on Egger’s tests in Stata MP 17, with a significance level set at *p* < 0.1 (Supplementary Table S1).

### Meta-analysis

A total of 12 studies were excluded from this meta-analysis due to the following reasons. Specifically, the outcomes of two studies (71, 73) were shown as bar graphs, and two (66, 74) were presented as line charts rather than specific values. Individual outcome indicators in four studies (56, 58, 67, 74) could not be integrated with other studies. Zheng’s (77) date was unacquirable. Data in three studies (62–64) of Zhu was described by Median and quartile which could not be counted. Finally, a total of 35 studies were included.

### Aphasia quotient

**Figure 1:**
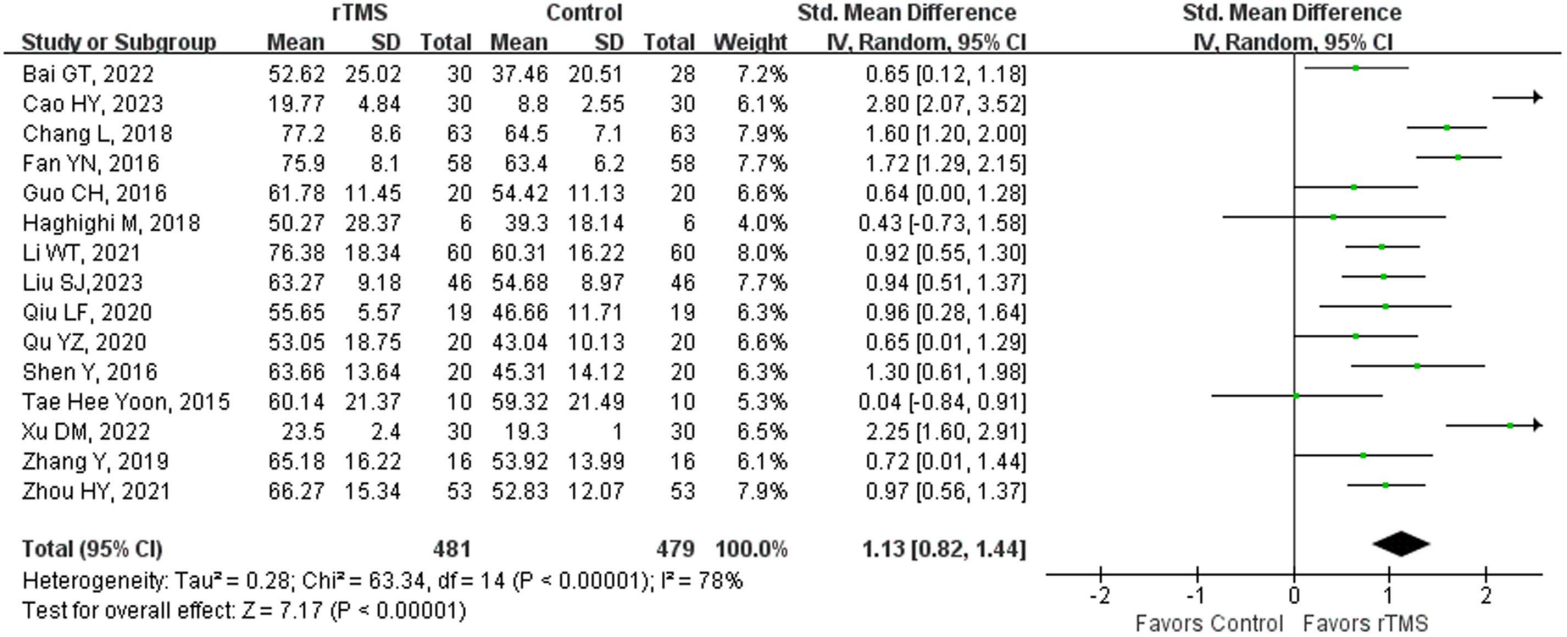
Aphasia quotient after rTMS.

**Figure 2:**
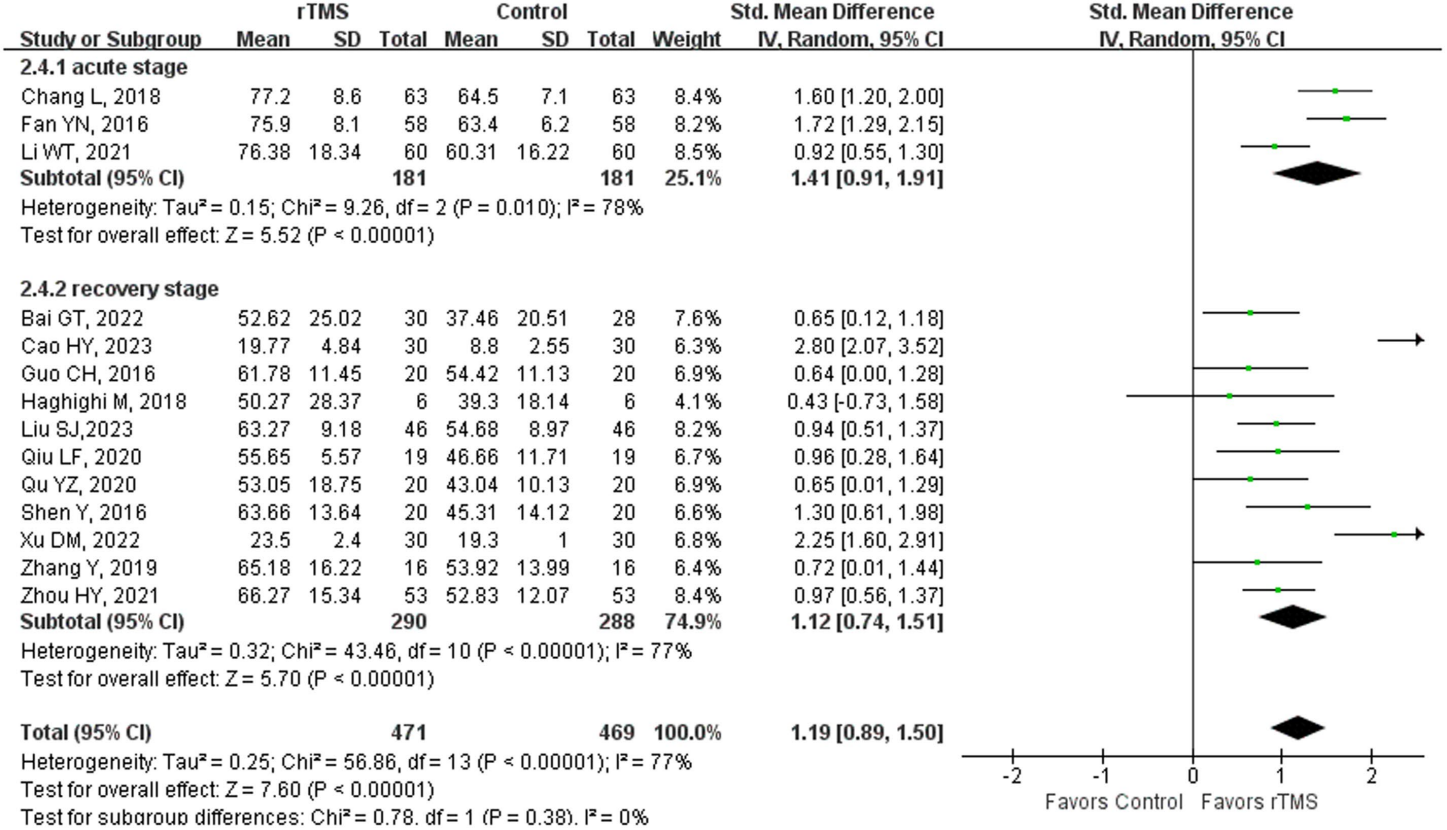
Aphasia quotient after rTMS subgroup analysis according to different stroke stages.

**Figure 3:**
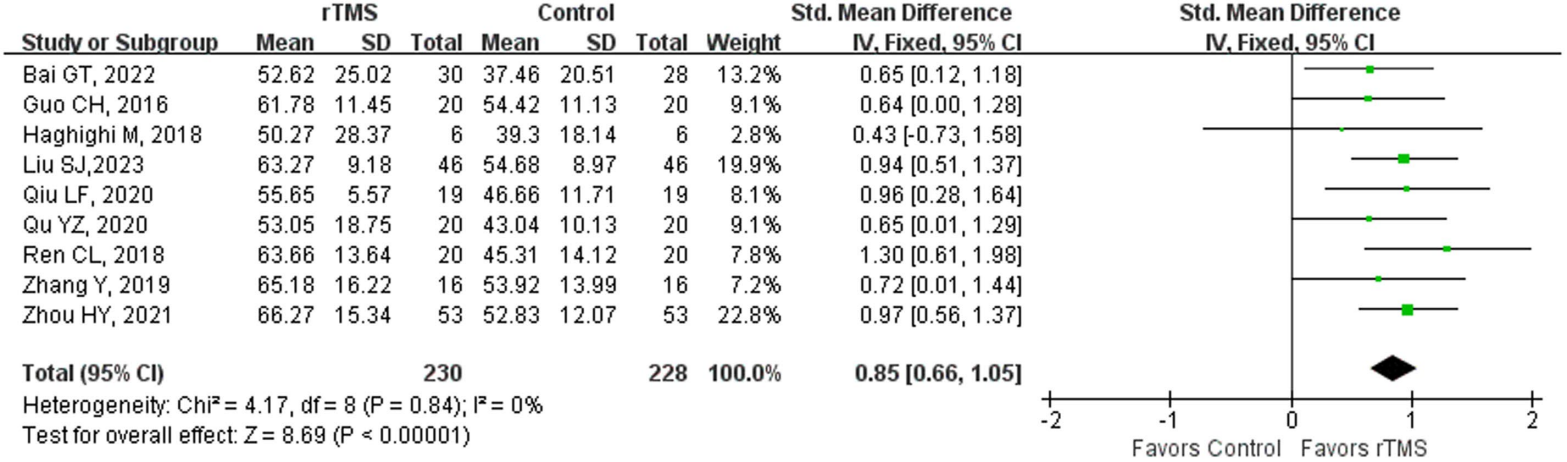
Aphasia quotient of stroke patients in the recovery stage (sensitivity analysis).

Fifteen studies (23, 36–38, 41, 42, 44, 46, 51, 52, 57, 59, 61, 65, 75) involving 960 patients assessed aphasia quotient (AQ) in patients after rTMS stimulation. The random-effects model was utilized due to significant heterogeneity across the studies (*I*^2^ = 78%). Global language ability of the rTMS-treated group exhibited a substantial improvement in comparison to the control group [*p* < 0.00001] (Figure 1). Subgroup analysis was conducted according to the stroke stages of patients after removing a study (50) with unidentified stroke duration and another study (75) that patients were in the sequelae stage. The random-effects model was employed for the following studies: three studies (37, 41, 44) with patients in the acute phase [SMD = 1.41, 95% CI (0.91, 1.91), *p* < 0.00001, *I*^2^ = 78%] and 11 studies (23, 36, 38, 42, 46, 51, 52, 57, 59, 61, 65) with patients in the recovery stage [*p* < 0.00001, *I*^2^ = 77%] (Figure 2). Upon conducting sensitivity analysis, the removal of two studies (36, 57) from the subgroup of recovery, the following outcomes were observed: 9 studies (23, 38, 42, 46, 51, 52, 59, 61, 65) of recovery subgroup [*p* < 0.00001, *I*^2^ = 0%] (Figure 3). The findings suggest that rTMS has the potential to enhance the overall language abilities of stroke patients during both acute and recovery phases.

### Repetition

**Figure 4:**
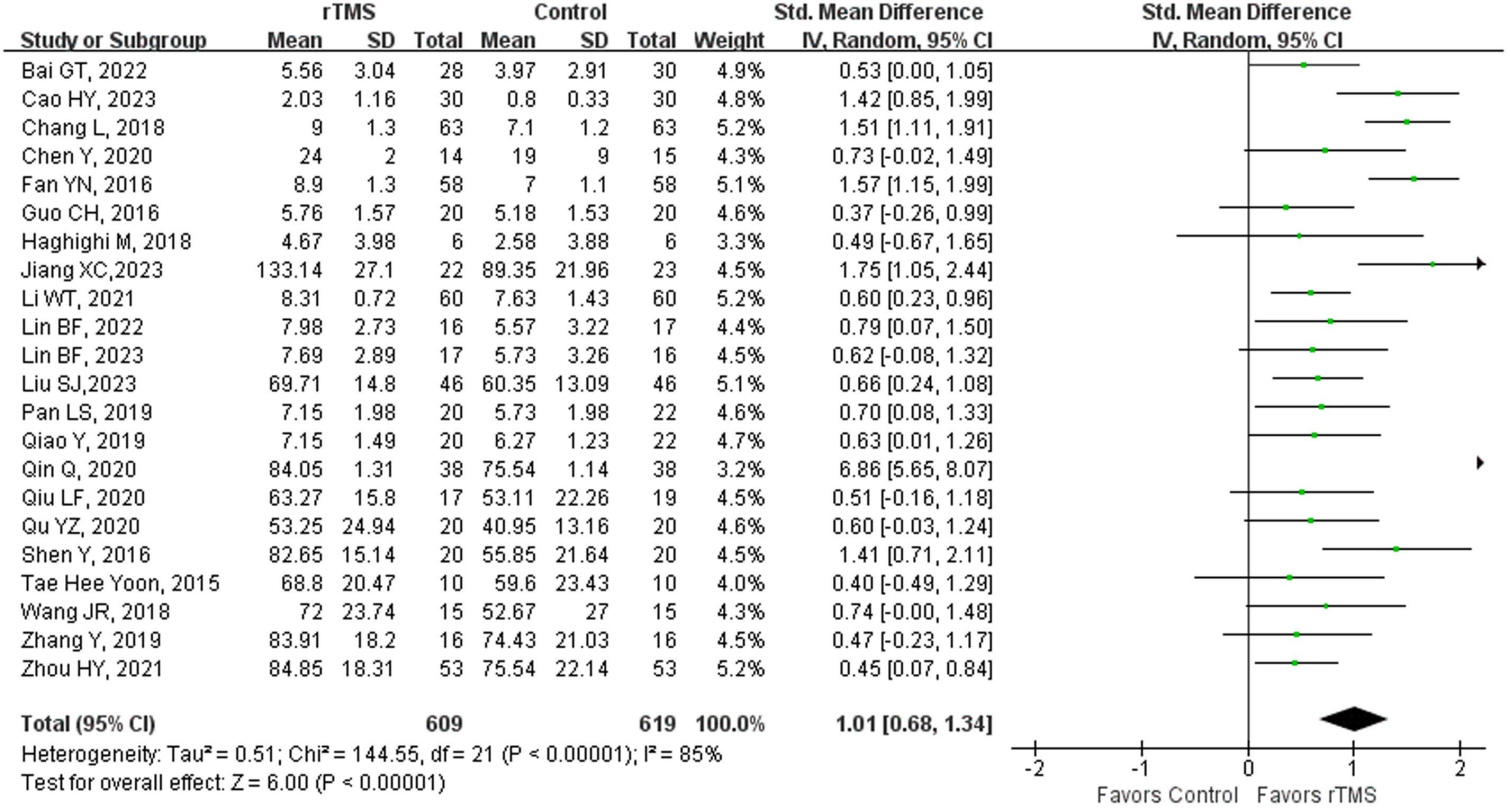
Repetition capability after rTMS.

**Figure 5:**
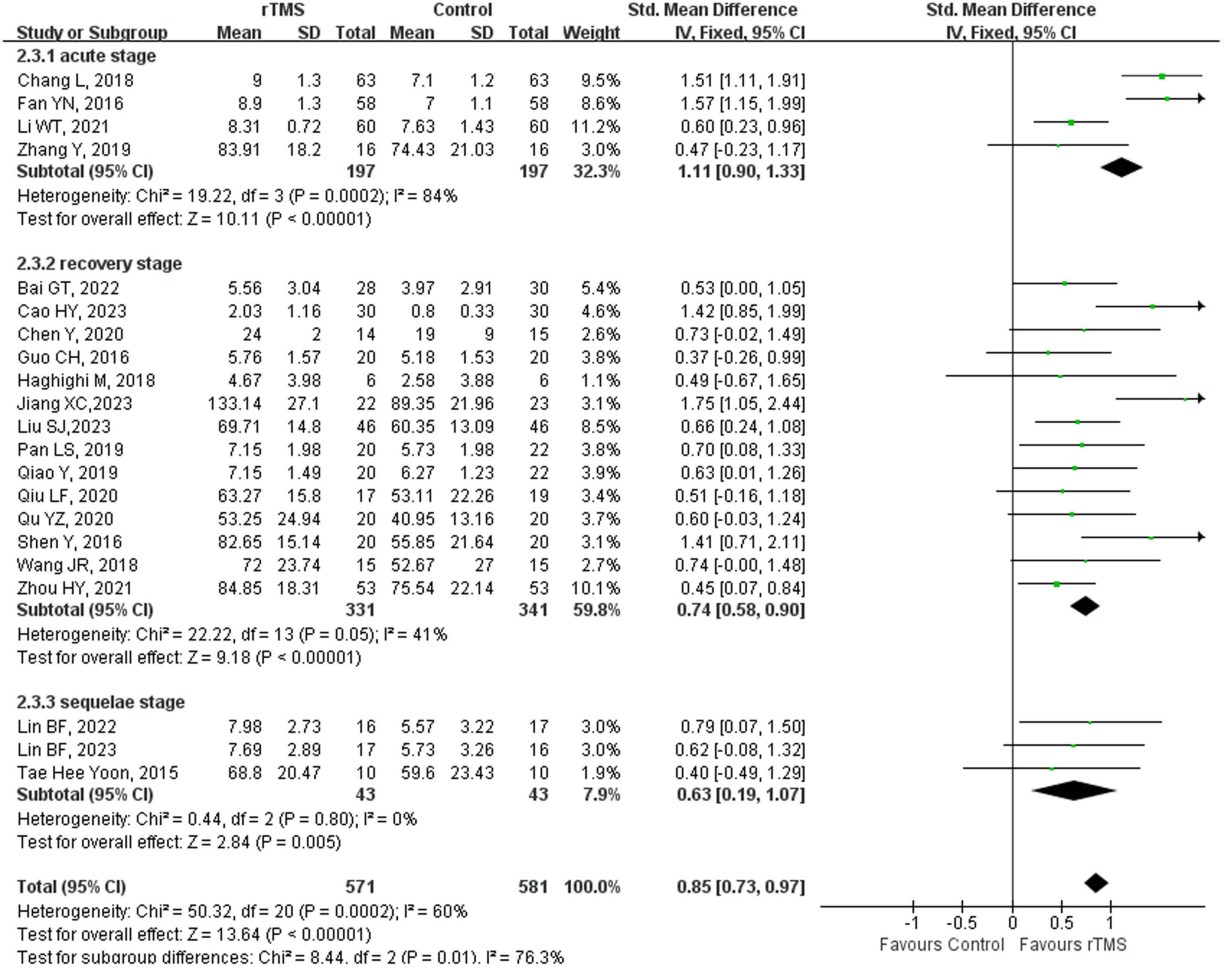
Repetition capability after rTMS subgroup analysis according to different stroke stages.

Twenty-two studies (23, 36–39, 41–44, 46, 48–52, 55, 59, 61, 65, 68, 69, 75) involving 1,228 patients assessed repetition ability in patients after rTMS treatment. The random-effects model was utilized due to significant heterogeneity across the studies (*I^2^* = 85%). Repetition ability of rTMS-treated group exhibited a substantial improvement in comparison to the control group [*p* < 0.00001] (Figure 4). Subgroup analysis was conducted according to the stroke stages of patients after removing a study (50) with unidentified stroke duration. The random-effects model was employed for the following studies: four studies (37, 41, 44, 59) with patients in the acute stage [*p* < 0.00001, *I^2^* = 84%]. The fixed-effects model was employed for the following studies: 14 studies (23, 36, 38, 39, 42, 43, 46, 48, 49, 51, 52, 55, 61, 65) with patients in the recovery stage [*p* < 0.00001, *I*^2^ = 41%] and three studies (68, 69, 75) with patients in the sequelae stage [*p* = 0.005, *I*^2^ = 0%] (Figure 5). These findings suggest that rTMS enhance repetition abilities in patients across various stages of stroke recovery. Notably, beyond immediate benefits, rTMS appears to exert medium- to long-term effects on language improvement. For individuals with NFA post-stroke, the positive effects of rTMS on speech enhancement persisted for up to 12 months (66).

### Naming

**Figure 6:**
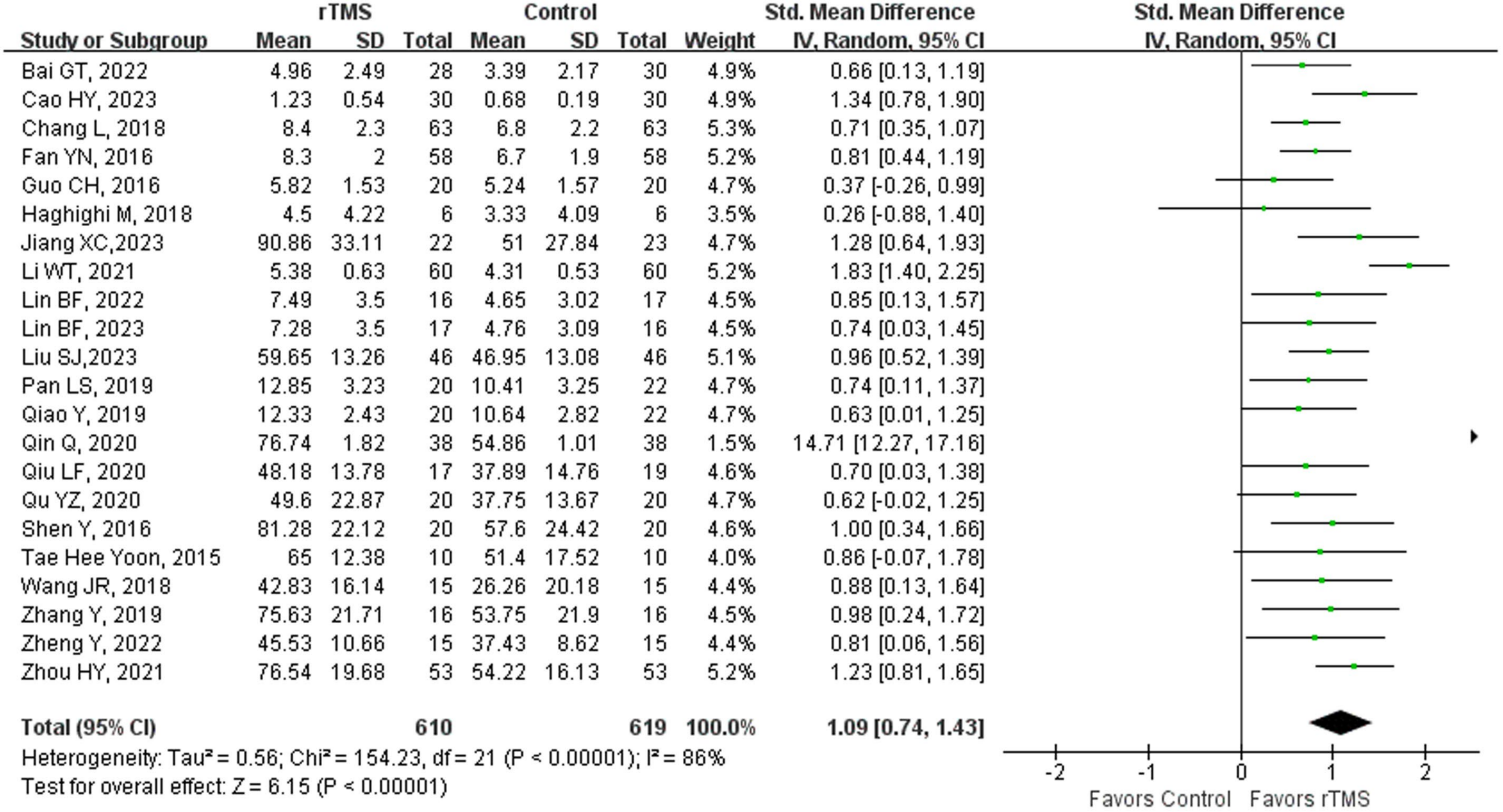
Naming capability after rTMS.

**Figure 7:**
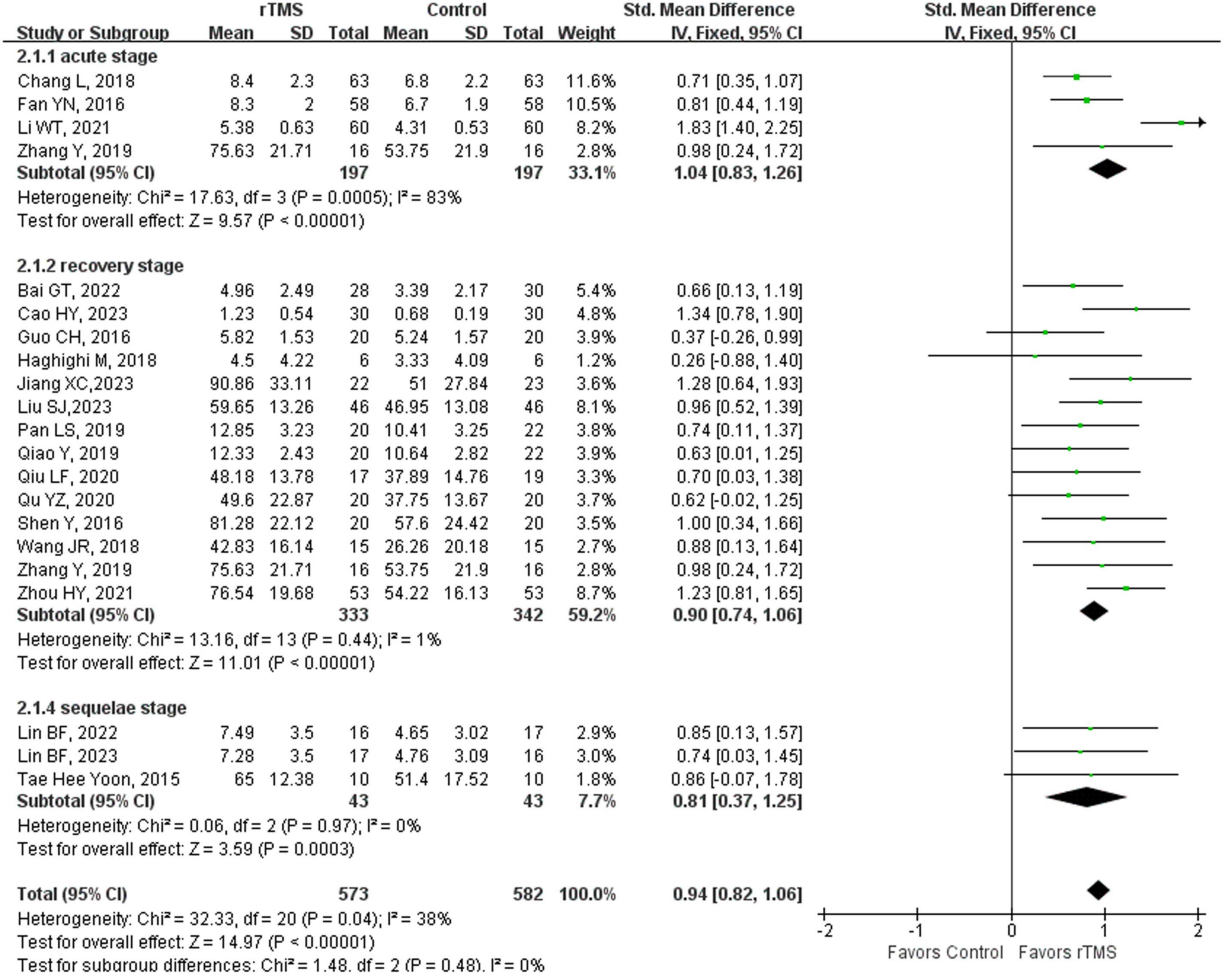
Naming capability after rTMS subgroup analysis according to different stroke stages.

Twenty-two (23, 36–38, 41–44, 46, 48–52, 55, 59–61, 65, 68, 69, 75) studies involving 1,229 patients evaluated naming ability in patients after rTMS. The random-effects model was utilized due to significant heterogeneity across the studies (*I*^2^ = 86%). Naming ability of the rTMS-treated group exhibited a substantial improvement in comparison to the control group [*p* < 0.00001] (Figure 6). Subgroup analysis was conducted according to the stroke stages of patients after removing a study (50) with unidentified stroke duration. The random-effects model was employed for the following studies: four studies (37, 41, 44, 59) with patients in the acute stage [*p* < 0.00001, *I*^2^ = 83%]. The fixed-effects model was employed for the following studies: 14 studies (23, 36, 38, 42, 43, 46, 48, 49, 51, 52, 55, 59, 61, 65) with patients in the recovery stage of [*p* < 0.00001, *I*^2^ = 1%] and three studies (68, 69, 75) with patients in the sequelae stage [*p* = 0.0003, *I*^2^ = 0%] (Figure 7). Naming is among the most challenging functions for stroke patients with NFA to regain. The restoration of naming capabilities necessitates the involvement of extensive neural networks, and rTMS has been shown to facilitate the recovery of these abilities across various stages of stroke, primarily by enhancing the connectivity among pertinent brain areas (65).

### Spontaneous language

**Figure 8:**
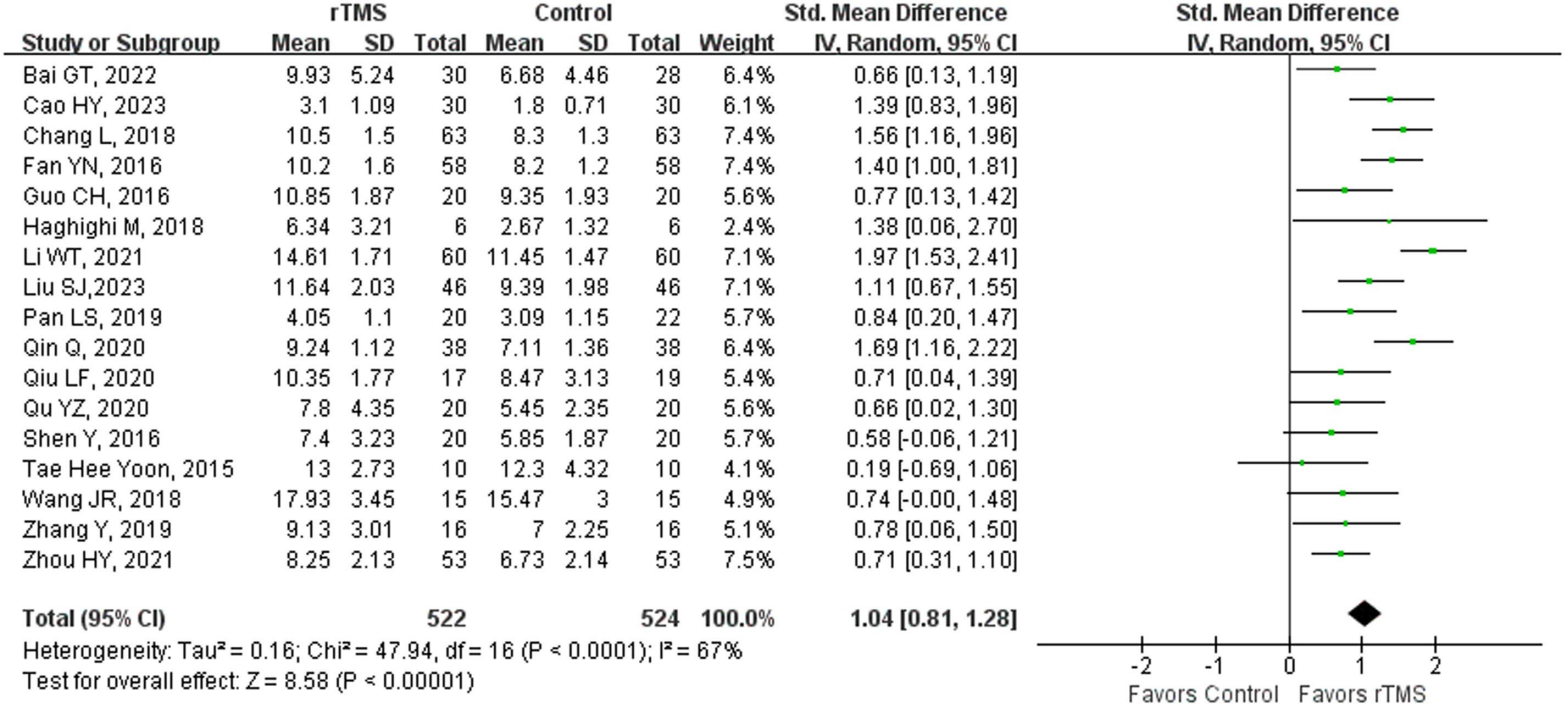
Spontaneous language capability after rTMS.

**Figure 9:**
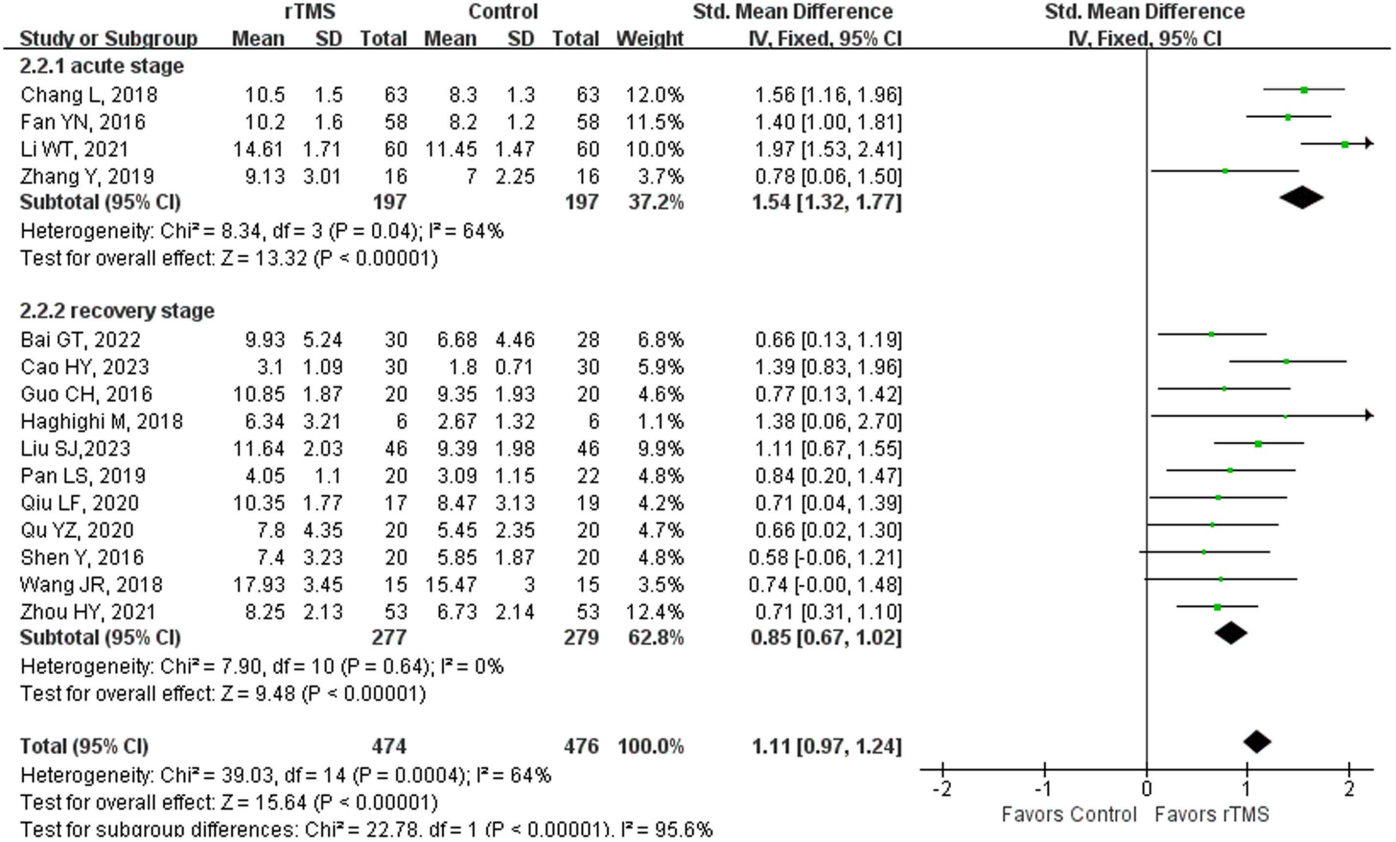
Spontaneous language capability after rTMS subgroup analysis according to different stroke stages.

Seventeen studies (4, 23, 36–38, 41, 42, 44, 46, 48, 50–52, 55, 59, 61, 65) involving 1,046 patients assessed spontaneous language ability in patients after rTMS stimulation. The random-effects model was utilized due to significant heterogeneity across the studies (*I^2^* = 67%). Spontaneous language ability of the rTMS-treated group exhibited a substantial improvement in comparison to the control group [*p* < 0.00001] (Figure 8). Subgroup analysis was conducted according to the stroke stages of patients after removing a study (50) with unidentified stroke duration and another study (75) that patients were in the sequelae stage. The random-effects model was employed for the following studies: four studies (37, 41, 44, 59) with patients in the acute stage [*p* < 0.00001, *I*^2^ = 64%]. The fixed-effects model was utilized for the following studies: 11 studies (23, 36, 38, 42, 46, 48, 51, 52, 55, 61, 65) with patients in the recovery stage [*p* < 0.00001, *I*^2^ = 0%] (Figure 9). These results suggested that rTMS ameliorated spontaneous language capability in patients in acute and recovery stages. Acupuncture therapy constitutes a significant component of complementary and alternative medicine, which has potential therapeutic effects in the treatment of PSA (81). The combined therapeutic application of acupuncture and rTMS on NFA has demonstrated a markedly superior efficacy compared to monotherapy. Specifically, the overall effectiveness of combining 1 Hz rTMS with acupuncture for NFA reached 96.66%, strongly associated with enhanced blood flow velocity and perfusion in the left middle cerebral artery (MCA) (44). Interaction between various acupuncture techniques and rTMS frequencies in producing distinct clinical outcomes warrants further investigation.

### BDNF

**Figure 10:**
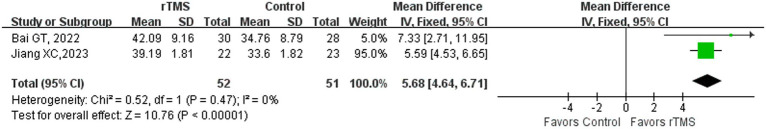
BDNF after rTMS.

Two studies (43, 65) involving 103 patients assessed the concentration of brain-derived neurotrophic factor (BDNF) within patients. The fixed-effects model was utilized due to low heterogeneity across the studies (*I*^2^ = 0%). The rTMS-treated group exhibited a substantial enhancement in serum BDNF concentration in comparison to the control group [*p* < 0.00001] (Figure 10). The findings indicated that rTMS has the potential to elevate the concentration of BDNF in the serum of stroke patients.

### Mood

**Figure 11:**
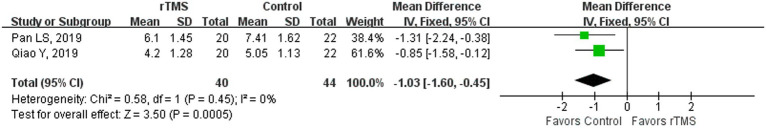
HAMD after rTMS.

Two studies (48, 49) involving 84 patients used the Hamilton Depression Scale (HAMD) to evaluate the effects of rTMS on mood in stroke patients. The fixed-effects model was utilized due to low heterogeneity across the studies (*I*^2^ = 0%). The HAMD score of rTMS group was notably lower in comparison to the control group [*p* = 0.0005] (Figure 11). The findings indicated that rTMS had potential to ameliorate the depression of these patients.

## Discussion

Numerous research groups, including those led by Naeser, Hamilton, Heiss, and Thiel, have showcased the effectiveness of rTMS in the treatment of PSA through significant studies. Despite their contributions, these studies were excluded from the review for various reasons. Hamilton’s research indicated that rTMS offers sustained enhancements in picture naming and fluency in patients with NFA. However, limitations included the absence of a control group in one study (82) and crossover of sham group participants to actual rTMS treatment in another (71). Heiss and Thiel observed a delayed beneficial impact of LF-rTMS on the right pars triangularis (R IFG pr) in enhancing naming ability in subacute PSA patients, yet these individuals were not suffering from NFA (83–87). The Naeser team reported improvements in Phrase Length and picture naming on the BNT following the suppression of the posterior R IFG pr through the application of LF-rTMS in NFA patients. Nevertheless, the exclusion was due to the absence of control groups not comprised of stroke patients, rendering it impossible to extract and cross-reference assessment data (88–93).

The present study aimed to provide an updated overview of the current evidence about the efficacy of rTMS in treating NFA. Firstly, this review found that rTMS could improve NFA in stroke patients, evidenced by increased aphasia quotient (AQ) scores in the rTMS group. AQ is an indicator of aphasia severity and serves as a metric for assessing aphasia improvement (94). Subgroup analysis revealed that rTMS significantly enhanced repetition and naming abilities in stroke patients at various stages. Additionally, spontaneous language improvement was noted in both acute and recovery phase patients, although the effects in the sequelae stage require further investigation. In our pursuit to examine studies focusing on the impact of rTMS on language skills, we discovered that several of these studies provided valuable insights beyond our initial scope. Interestingly, this exploration led us to understand that depression alleviation and the increase of BDNF may also benefit from rTMS. Admittedly, these findings are only based on 2 studies (each), and further research is necessary.

Stroke damages brain regions responsible for language expression and auditory comprehension, leading to aphasia, which in turn worsens functional outcomes (95). Aphasia improvement is linked to the rebalancing of activity between the perilesional ipsilateral and contralateral hemispheres, making rTMS a promising method for promoting language recovery (5, 96). The meta-analysis primarily focused on single-site and LF-rTMS stimulation, uncovering a notable association between the activation level of the IFG-R and patients’ fluency (97). Lefaucheur et al. (16) proposed Level B evidence supporting the utilization of LF-rTMS on the IFG-R in patients suffering from NFA, especially when combined with SLT. Both HF-rTMS and LF-rTMS applied to one hemisphere, have demonstrated effectiveness in treating NFA (13). HF-rTMS enhances cerebral cortex excitability and revives bilateral cerebral hemisphere function by stimulating local neurons in the language center (98). However, HF-rTMS can cause intracranial hemorrhage and epilepsy, leading to its limited use in clinical and research settings. In one study (67), bilateral hemispheric stimulation (LF-rTMS applied to the right unaffected Broca’s area and HF-rTMS targeting the left affected Broca’s area) led to significant language function improvements, including repetition, naming, word comprehension, and fluency. These improvements were observed immediately after treatment and lasted for 2 months. Similarly, Vuksanović et al. (99) observed that the integration of cTBS applied to the right hemisphere with iTBS targeting the left, followed by 45 min of SLT, improved various language functions.

Aphasia, a neural network disorder, involves changes in the brain’s functional connections. The reinstatement of the language network structure and function is crucial for restoring language abilities in individuals with aphasia (100). Regional homogeneity (Reho) analysis revealed that in aphasia patients, the activation of the IFG-L and left cuneiform lobe was lower compared to normal subjects, and there was a reduction in the functional connection between the left medial temporal gyrus (MTG-L) and superior temporal gyrus (STG-L) (101). rTMS has been demonstrated to enhance the functional connections between the bilateral frontal lobes and the left temporal lobe (35). Lin et al. (69) investigated the relationship between functional connectivity in language-related regions and language performance. The LF-rTMS group demonstrated significant functional connectivity remodeling, which fostered positive changes in brain plasticity. This aligns with the theory of improving the “Inter-hemispheric competition pattern” (102). Therefore, imaging analysis holds a crucial role in comprehensively assessing disruption and remodeling of the language network following a stroke.

Regarding the risk of bias, Egger’s test results indicated that publication bias was not significant in the language domains such as repetition and overall language proficiency (*p* > 0.1). However, there was a significant presence of bias in naming and spontaneous speech categories (*p* < 0.1). Of the seven domains assessed by RevMan 5.4, the domain of random sequence generation exhibited the highest risk of bias.

The review also indicated that rTMS can enhance linguistic functions by increasing serum BDNF levels. BDNF, the most abundant neurotrophin in the cerebrum and predominantly found in the forebrain, is closely associated with cognitive and language functions. It plays a crucial role in facilitating the neuroplasticity process in PSA patients (65). LF-rTMS (43) and iTBS (65) have been shown to elevate BDNF levels in the peripheral serum of the rTMS group, reflecting changes in brain BDNF concentration. Post-stroke depression (PSD) affects 30–60% of stroke survivors (103), which also relates to the language performance of stroke patients (10). Recognized as an effective treatment for depression, rTMS is supported by level A evidence (16). The meta-analysis demonstrated that both LF-rTMS and HF-rTMS significantly reduced depression scores and improved mood in the meta-analysis. Our findings indicate that lower HAMD scores correlate with better linguistic function performance. In conclusion, notable improvements in mood or serum BDNF from rTMS in NFA patients positively influence linguistic functions.

### Limitations and future directions

Recognition of numerous constraints is essential in this systematic review. Firstly, the high proportion of Chinese literature may cause some publication bias. Secondly, clinical and methodological heterogeneity among the included literature may influence overall results, including the age of patients and varied rTMS treatment regimens. Thirdly, owing to restrictions on article length, only some meta-analysis findings were reported. In the future, we can discuss the therapeutic effects of different dosages of rTMS and different rTMS approaches, and design accurate rTMS parameters according to aphasia types, combined with functional imaging technology to further explore the related mechanisms.

## Conclusion

Collectively, the reviewed literature provides compelling support for the utilization of rTMS as a viable non-pharmacological intervention aiding in the recovery of non-fluent aphasia post-stroke, including the ability of repetition, naming, and spontaneous language which may be accompanied by the improvement of serum BDNF and alleviation in depression in patients.

## Data availability statement

The original contributions presented in the study are included in the article/Supplementary material, further inquiries can be directed to the corresponding author.

## Author contributions

JC: Writing – original draft. YJ: Methodology, Writing – review & editing, Formal analysis. TR: Data curation, Writing – review & editing, Software. YY: Supervision, Writing – review & editing. YL: Supervision, Writing – review & editing. YZ: Writing – review & editing. SY: Writing – review & editing, Funding acquisition, Visualization.

## References

[1] Greco A Occhipinti G Giacoppo D Agnello F Laudani C Spagnolo M . Antithrombotic therapy for primary and secondary prevention of ischemic stroke: JACC state-of-the-art review. J Am Coll Cardiol. (2023) 82:1538–57. doi: 10.1016/j.jacc.2023.07.025, PMID: 37793752

[2] Zhang Y He X Hu S Hu S He F Shen Y . Efficacy and safety of massage in the treatment of post-stroke insomnia: a protocol for systematic review and meta-analysis. Medicine. (2020) 99:e23598. doi: 10.1097/MD.0000000000023598, PMID: 33371092 PMC7748325

[3] Vitti E Hillis AE. Treatment of post-stroke aphasia: a narrative review for stroke neurologists. Int J Stroke. (2021) 16:1002–8. doi: 10.1177/17474930211017807, PMID: 33949274 PMC8645656

[4] Low TA Lindland K Kirton A Carlson HL Harris AD Goodyear BG . Repetitive transcranial magnetic stimulation (RTMS) combined with multi-modality aphasia therapy for chronic post-stroke non-fluent aphasia: a pilot randomized sham-controlled trial. Brain Lang. (2023) 236:105216. doi: 10.1016/j.bandl.2022.105216, PMID: 36525719

[5] Crosson B Rodriguez AD Copland D Fridriksson J Krishnamurthy LC Meinzer M . Neuroplasticity and aphasia treatments: new approaches for an old problem. J Neurol Neurosurg Psychiatry. (2019) 90:1147–55. doi: 10.1136/jnnp-2018-319649, PMID: 31055282 PMC8014302

[6] Georgiou AM Kambanaros M. The effectiveness of transcranial magnetic stimulation (TMS) paradigms as treatment options for recovery of language deficits in chronic Poststroke aphasia. Behav Neurol. (2022) 2022:1–25. doi: 10.1155/2022/7274115 PMC8767406 35069929

[7] Poslawsky IE Schuurmans MJ Lindeman E Hafsteinsdóttir TB. A systematic review of nursing rehabilitation of stroke patients with aphasia. J Clin Nurs. (2010) 19:17–32. doi: 10.1111/j.1365-2702.2009.03023.x, PMID: 20500241

[8] Sinanović O Mrkonjić Z Zukić S Vidović M Imamović K. Post-stroke language disorders. Acta Clin Croat. (2011) 50:79–94. PMID: 22034787

[9] Bonilha L Hillis AE Wilmskoetter J Hickok G Basilakos A Munsell B . Neural structures supporting spontaneous and assisted (entrained) speech fluency. Brain. (2019) 142:3951–62. doi: 10.1093/brain/awz309, PMID: 31580418 PMC6885692

[10] Sheppard SM Sebastian R. Diagnosing and managing post-stroke aphasia. Expert Rev Neurother. (2021) 21:221–34. doi: 10.1080/14737175.2020.1855976, PMID: 33231117 PMC7880889

[11] Haro-Martínez A Pérez-Araujo CM Sanchez-Caro JM Fuentes B Díez-Tejedor E. Melodic intonation therapy for post-stroke non-fluent aphasia: systematic review and Meta-analysis. Front Neurol. (2021) 12:700115. doi: 10.3389/fneur.2021.700115, PMID: 34421802 PMC8371046

[12] Marchina S Norton A Schlaug G. Effects of melodic intonation therapy in patients with chronic nonfluent aphasia. Ann N Y Acad Sci. (2023) 1519:173–85. doi: 10.1111/nyas.14927, PMID: 36349876 PMC10262915

[13] Starosta M Cichoń N Saluk-Bijak J Miller E. Benefits from repetitive transcranial magnetic stimulation in post-stroke rehabilitation. J Clin Med. (2022) 11:2149. doi: 10.3390/jcm11082149, PMID: 35456245 PMC9030945

[14] Hara T Shanmugalingam A McIntyre A Burhan AM. The effect of non-invasive brain stimulation (nibs) on attention and memory function in stroke rehabilitation patients: a systematic review and Meta-analysis. Diagnostics. (2021) 11:227. doi: 10.3390/diagnostics11020227, PMID: 33546266 PMC7913379

[15] Kesikburun S . Non-invasive brain stimulation in rehabilitation. Turk J Phys Med Rehabil. (2022) 68:1–8. doi: 10.5606/tftrd.2022.10608, PMID: 35949977 PMC9305642

[16] Lefaucheur JP Aleman A Baeken C Benninger DH Brunelin J di Lazzaro V . Evidence-based guidelines on the therapeutic use of repetitive transcranial magnetic stimulation (RTMS): an update (2014-2018). Clin Neurophysiol. (2020) 131:474–528. doi: 10.1016/j.clinph.2019.11.002, PMID: 31901449

[17] Martin PI Naeser MA Theoret H Tormos JM Nicholas M Kurland J . Transcranial magnetic stimulation as a complementary treatment for aphasia. Semin Speech Lang. (2004) 25:181–91. doi: 10.1055/s-2004-825654, PMID: 15118944

[18] Chou TY Wang JC Lin MY Tsai PY. Low-frequency vs. Theta burst transcranial magnetic stimulation for the treatment of chronic non-fluent aphasia in stroke: a proof-of-concept study. Front Aging Neurosci. (2022) 13:800377. doi: 10.3389/fnagi.2021.800377, PMID: 35095477 PMC8795082

[19] Fahmy EM Elshebawy HM. Effect of high frequency transcranial magnetic stimulation on recovery of chronic post-stroke aphasia. J Stroke Cerebrovasc Dis. (2021) 30:105855. doi: 10.1016/j.jstrokecerebrovasdis.2021.105855, PMID: 34049013

[20] Georgiou A Konstantinou N Phinikettos I Kambanaros M. Neuronavigated theta burst stimulation for chronic aphasia: two exploratory case studies. Clin Linguist Phon. (2019) 33:532–46. doi: 10.1080/02699206.2018.1562496, PMID: 30676091

[21] Ren C Zhang G Xu X Hao J Fang H Chen P . The effect of RTMS over the different targets on language recovery in stroke patients with global aphasia: a randomized sham-controlled study. Biomed Res Int. (2019) 2019:1–7. doi: 10.1155/2019/4589056 PMC6699349 31467892

[22] Hu XY Zhang T Rajah GB Stone C Liu LX He JJ . Effects of different frequencies of repetitive transcranial magnetic stimulation in stroke patients with non-fluent aphasia: a randomized, sham-controlled study. Neurol Res. (2018) 40:459–65. doi: 10.1080/01616412.2018.1453980, PMID: 29589518

[23] Haghighi M Mazdeh M Ranjbar N Seifrabie MA. Further evidence of the positive influence of repetitive transcranial magnetic stimulation on speech and language in patients with aphasia after stroke: results from a double-blind intervention with sham condition. Neuropsychobiology. (2017) 75:185–92. doi: 10.1159/000486144, PMID: 29402816

[24] Hebert D Lindsay MP McIntyre A Kirton A Rumney PG Bagg S . Canadian stroke best practice recommendations: stroke rehabilitation practice guidelines, update 2015. Int J Stroke. (2016) 11:459–84. doi: 10.1177/1747493016643553, PMID: 27079654

[25] Zhang J Zhong D Xiao X Yuan L Li Y Zheng Y . Effects of repetitive transcranial magnetic stimulation (rtms) on aphasia in stroke patients: a systematic review and meta-analysis. Clin Rehabil. (2021) 35:1103–16. doi: 10.1177/0269215521999554, PMID: 33706572

[26] Arheix-Parras S Barrios C Python G Cogné M Sibon I Engelhardt M . A systematic review of repetitive transcranial magnetic stimulation in aphasia rehabilitation: leads for future studies. Neurosci Biobehav Rev. (2021) 127:212–41. doi: 10.1016/j.neubiorev.2021.04.008, PMID: 33862065

[27] Gholami M Pourbaghi N Taghvatalab S. Evaluation of rtms in patients with poststroke aphasia: a systematic review and focused meta-analysis. Neurol Sci. (2022) 43:4685–94. doi: 10.1007/s10072-022-06092-x, PMID: 35499630

[28] Kielar A Patterson D Chou YH. Efficacy of repetitive transcranial magnetic stimulation in treating stroke aphasia: systematic review and meta-analysis. Clin Neurophysiol. (2022) 140:196–227. doi: 10.1016/j.clinph.2022.04.017, PMID: 35606322

[29] Li T Zeng X Lin L Xian T Chen Z. Effects of repetitive transcranial magnetic stimulation with different frequencies on post-stroke aphasia: a Prisma-compliant meta-analysis. Medicine. (2020) 99:e20439. doi: 10.1097/MD.0000000000020439, PMID: 32541465 PMC7302648

[30] Wang C Nie P Wang P Wang Y Zang Y Zhang Y. The therapeutic effect of transcranial magnetic stimulation on post-stroke aphasia and the optimal treatment parameters: a meta-analysis. Arch Phys Med Rehabil. (2023). doi: 10.1016/j.apmr.2023.11.006, PMID: 37984539

[31] Georgiou AM Lada E Kambanaros M. Evaluating the quality of conduct of systematic reviews on the application of transcranial magnetic stimulation (TMS) for post-stroke aphasia rehabilitation. Aphasiology. (2019) 34:540–56. doi: 10.1080/02687038.2019.1632786

[32] Zheng Y Liu J Wang Y Xing J Gu Y Tang J. Low-frequency repetitive transcranial magnetic stimulation for non-fluent aphasia after stroke: a systematic review. Chin J Rehabil. (2021) 36:365–71.

[33] Page MJ McKenzie J Bossuyt PM Boutron I Hoffmann TC Mulrow CD . The Prisma 2020 statement: an updated guideline for reporting systematic reviews. BMJ. (2021) 372:n71. doi: 10.1136/bmj.n71, PMID: 33782057 PMC8005924

[34] Sterne JAC Savović J Page MJ Elbers RG Blencowe NS Boutron I . RoB 2: a revised tool for assessing risk of bias in randomised trials. BMJ. (2019) 366:l4898. doi: 10.1136/bmj.l4898 31462531

[35] Bai G Jiang L Sun D Meng P Yang CH Zhang Z . Based on the regional homogeneity method to explore the effect and mechanism of low-frequency repetitive transcranial magnetic stimulation on the auditory comprehension function of post- stroke patients with non-fluent aphasia. Chin J Rehabil Med. (2023) 38:606–12. doi: 10.3969/j.issn.1001-1242.2023.05.004

[36] Cao H Zhang W Xu T Cui X. Effect of eye tracking training combined with repetitive transcranial magnetic stimulation on post-stroke complete aphasia. Chin J Rehabil. (2023) 38:552–5. doi: 10.3870/zgkf.2023.09.009

[37] Chang L . Clinical observation of low frequency repetitive transcranial magnetic stimulation on motor aphasia after acute cerebral infarction. Neural Injury Funct Reconstr. (2018) 13:53–4. doi: 10.16780/j.cnki.sjssgncj.2018.01.020

[38] Shen Y Yin Z Zhou Q Cong F Yi W Shan CH. Low frequency, repetitive transcranial magnetic stimulation can alleviate non-fluent aphasia after stroke. Chin J Phys Med Rehabil. (2016) 3:170–4. doi: 10.3760/cma.j.issn.0254-1424.2016.03.003

[39] Chen Y Gao H Wang X Zhou Y Ou X Pan C. Effect of low-frequency RTMS on rehabilitation of language and communication ability in patients with non-fluent aphasia after stroke. Chin J Gerontol. (2020) 40:709–12. doi: 10.3969/j.issn.1005-9202.2020.04.011

[40] Shan Y Wang L Wang J Luo Z Zeng X. The effect of low frequency transcranial magnetic stimulation on aphasia after cerebral infarction. Chin J Phys Med Rehabil. (2012) 5:361–4. doi: 10.3760/cma.j.issn.0254-1424.2012.05.01

[41] Fan Y Zhao J. Therapeutic effect of low-frequency repetitive transcranial magnetic stimulation on motor aphasia after acute cerebral infarction. Chin J Rehabil. (2016) 31:28–30. doi: 10.3870/zgkf.2016.01.008

[42] Guo C Zhu G Deng W Ma T Du A Zhao Z. Effect of repetitive transcranial magnetic stimulation combined with memantine hydrochloride and speech therapy on the treatment of motor aphasia after cerebral infarction. China Med Herald. (2016) 13:83–6.

[43] Jiang X Liu Z Su Q Zhao Q Xia X Lu F. Effect of intermittent theta burst transcranial magnetic stimulation on non-fluent aphasia after stroke. Chin J Rehabil Theory Pract. (2023) 29:839–43. doi: 10.3969/j.issn.1006.9771.2023.07.014

[44] Li W Qin H Wei X Zhao P. Clinical observation on the treatment of post-stroke motor aphasia with consciousness-restoring resuscitation acupuncture combined with transcranial magnetic stimulation. J Pract Trad Chin Med. (2021) 37:858–60.

[45] Li Z Zhao Y Ren C Cai D Wu SH Fang H. Mechanism in the treatment of subacute motor aphasia with low frequency repetitive transcranial magnetic stimulation by quantitative electroencephalography. Chin J Rehabil Med. (2018) 33:794–9. doi: 10.3969/j.issn.1001-1242.2018.07.008

[46] Liu S Lv M Wang Y Liang J Li T. Contralateral inhibitory repetitive transcranial magnetic stimulation combined with donepezil in the treatment of post-stroke non-fluent aphasia. J Audiol Speech Pathol. (2023) 31:165–9. doi: 10.3969/j.issn.1006-7299.2023.02.016

[47] Liu S Hu X. Clinical observation of acupuncture combined with repetitive transcranial magnetic stimulation in the treatment of motor aphasia after stroke. Clin J Trad Chin Med. (2022) 34:2146–50. doi: 10.16448/j.cjtcm.2022.1135

[48] Pan L Ma J Peng Y Sun J Zhu B. Electro-acupuncture combined with high frequency repetitive transcranial magnetic stimulation in the treatment of motor aphasia after stroke. China J Chin Med. (2019) 34:1985–9. doi: 10.16368/j.issn.1674-8999.2019.09.463

[49] Qiao Y Ma J Peng Y Sun J Zhu B. Clinical study of electro-acupuncture combined with Low- frequency rtms on motor aphasia after stroke. J Clin Acupunct Moxibust. (2019) 35:15–9. doi: 10.3969/j.issn.1005-0779.2019.10.005

[50] Qin Q . Efficacy of low-frequency repetitive transcranial magnetic stimulation combined with hyperbaric medicine aphasia after stroke. Med Diet Health. (2020) 18:53–4.

[51] Qiu L Cai Y Jiang Y Lin W Li X. Effects of response elaboration training combined with RTMS in patients with non-fluent aphasia. Chin J Rehabil Med. (2020) 35:1192–7. doi: 10.3969/j.issn.1001-1242.2020.10.007

[52] Qu Y Zhong G Lv J . Observation on the efficacy of low frequency repetitive transcranial magnetic stimulation in the treatment of non-fluent aphasia after subacute stroke. China Modern Doctor. (2020) 58:112–5.

[53] Ren C Cai D Fang H Chen P Li ZH Wu SH. Effects of low frequency rtms over the right superior temporal gyrus on language function in subacute global poststroke aphasia patients. J Rehabil Med. (2018) 33:1055–9. doi: 10.3969/j.issn.1001-1242.2018.09.009

[54] Wang G Liu M Sheng Y He SH Xie J Long J. Clinical study of RTMS combined with speech training in the treatment of aphasia after stroke. Shenzhen J Integr Trad Chin Western Med. (2021) 31:1–4. doi: 10.16458/j.cnki.1007-0893.2021.06.001

[55] Wang J Li J Zhang Y Bai G Han CH Yang CH . Effect of low frequency repetitive transcranial magnetic stimulation on non-fluent aphasia after stroke. Chin J Rehabil Med. (2018) 33:1463–4. doi: 10.3969/j.issn.1001-1242.2018.12.018

[56] Wu G Yang H Pan L. Effect and mechanism of repetitive transcranial magnetic stimulation on aphasia in patients with left hemisphere cerebral infarction. Shandong Med J. (2017) 57:65–7. doi: 10.3969/j.issn.1002-266X.2017.37.022

[57] Xu D Liu H. Effect of repetitive transcranial magnetic stimulation combined with speech training on motor aphasia after stroke. Med Innov China. (2022) 19:147–50. doi: 10.3969/j.issn.1674-4985.2022.02.037

[58] Zhang D Wang J Lu J Wang P Cheng Y Yuan Y . Efficacy comparison of noninvasive brain stimulation techniques on picture naming ability of non fluent aphasia patients after stroke with normal expression capacity. Chin J Cerebrovasc Dis. (2021) 18:84–90. doi: 10.3969/j.issn.1672-5921.2021.02.002

[59] Zhang Y Yun W Zhang M Chen Y Cao Y Zhou X. The effects of low-frequency repetitive transcranial magnetic stimulation combined with hyperbaric oxygen in the treatment of non-fluent aphasia. Chin J Phys Med Rehabil. (2019) 7:512–6. doi: 10.3760/cma.j.issn.0254-1424.2019.07.008

[60] Zheng Y Gu Y Yuan L Zhang W Shi CH Tang J. Attention should be paid to the effect of training combined with repetitive transcranial magnetic stimulation in the treatment of non-fluent aphasia after stroke. J Audiol Speech Pathol. (2022) 30:304–7. doi: 10.3969/j.issn.1006-7229.2022.03.015

[61] Zhou H Yuan L Wen Y Jiang W Yang L Chen H . Effect of low-frequency repetitive transcranial magnetic stimulation combined with speech training on the rehabilitation of stroke aphasia. Neural Inj Funct Reconstr. (2021) 16:614–6. doi: 10.16780/j.cnki.sjssgncj.20201268

[62] Zhu H Cheng X Liu L Tian L Rao J Liu Y . Clinical observation of inhibitory repetitive transcranial magnetic stimulation in stroke patients with non-fluent aphasia. Zhejiang Med J. (2023) 45:1140–1145+1151. doi: 10.12056/j.issn.1006-2785.2023.45.11.2022-232

[63] Zhu H Zhang X Cheng X Rao J Zhang Y Liu L. Effect of inhibitory repetitive transcranial magnetic stimulation combined with mirror neuron training system for stroke patients with global aphasia. Chin J Rehabil. (2020) 35:563–7. doi: 10.3870/zgkf.2020.22.001

[64] Zhu H Zhang X Cheng X Rao J Zhang Y Liu L. The effect of inhibitory repetitive transcranial magnetic stimulation combined with Mirror neuron training system on chronic global aphasia Poststroke. Chin J Stroke. (2021) 16:45–50. doi: 10.3969/j.issn.1673-5765.2021.01.008

[65] Bai G Jiang L Huan S Meng P Wang Y Pan X . Study on Low-frequency repetitive transcranial magnetic stimulation improves speech function and mechanism in patients with non-fluent aphasia after stroke. Front Aging Neurosci. (2022) 14:883542. doi: 10.3389/fnagi.2022.883542, PMID: 35711903 PMC9197107

[66] Barwood CH Murdoch BE Riek S O'Sullivan JD Wong A Lloyd D . Long term language recovery subsequent to low frequency rtms in chronic non-fluent aphasia. NeuroRehabilitation. (2013) 32:915–28. doi: 10.3233/NRE-130915, PMID: 23867417

[67] Khedr EM Abo el-Fetoh N Ali AM el-Hammady DH Khalifa H Atta H . Dual-hemisphere repetitive transcranial magnetic stimulation for rehabilitation of poststroke aphasia: a randomized, double-blind clinical trial. Neurorehabil Neural Repair. (2014) 28:740–50. doi: 10.1177/1545968314521009, PMID: 24503205

[68] Lin BF Hon F Lin MY Tsai PY Lu CF. Right arcuate fasciculus as outcome predictor after low-frequency repetitive transcranial magnetic stimulation in nonfluent aphasic stroke. Eur J Neurol. (2023) 30:2031–41. doi: 10.1111/ene.15808, PMID: 36997303

[69] Lin BF Yeh SC Kao YJ Lu CF Tsai PY. Functional remodeling associated with language recovery after repetitive transcranial magnetic stimulation in chronic aphasic stroke. Front Neurol. (2022) 13:809843. doi: 10.3389/fneur.2022.809843, PMID: 35330805 PMC8940300

[70] Lopez-Romero LA Riano-Carreno DM Pachon-Poveda MY Mendoza-Sanchez JA Leon-Vargas YK Moreno-Pabon A . Efficacy and safety of transcranial magnetic stimulation in patients with non-fluent aphasia, following an ischaemic stroke. A controlled, randomised and double-blind clinical trial. Rev Neurol. (2019) 68:241–9. doi: 10.33588/rn.6806.2018300, PMID: 30855708

[71] Medina J Norise C Faseyitan O Coslett HB Turkeltaub PE Hamilton RH. Finding the right words: transcranial magnetic stimulation improves discourse productivity in non-fluent aphasia after stroke. Aphasiology. (2012) 26:1153–68. doi: 10.1080/02687038.2012.710316, PMID: 23280015 PMC3532848

[72] Tsai PY Wang CP Ko JS Chung YM Chang YW Wang JX. The persistent and broadly modulating effect of inhibitory rtms in nonfluent aphasic patients: a sham-controlled, double-blind study. Neurorehabil Neural Repair. (2014) 28:779–87. doi: 10.1177/1545968314522710, PMID: 24526709

[73] Wang CP Hsieh CY Tsai PY Wang CT Lin FG Chan RC. Efficacy of synchronous verbal training during repetitive transcranial magnetic stimulation in patients with chronic aphasia. Stroke. (2014) 45:3656–62. doi: 10.1161/STROKEAHA.114.007058, PMID: 25378426

[74] YAŞA İC MAVİŞ İ ŞALÇİNİ C MİDİ İ. Comparing the efficiency of speech and language therapy and transcranial magnetic stimulation for treating Broca's aphasia. J Stroke Cerebrovasc Dis. (2023) 32:107108. doi: 10.1016/j.jstrokecerebrovasdis.2023.107108, PMID: 37068324

[75] Yoon TH Han SJ Yoon TS Kim JS Yi TI. Therapeutic effect of repetitive magnetic stimulation combined with speech and language therapy in post-stroke non-fluent aphasia. NeuroRehabilitation. (2015) 36:107–14. doi: 10.3233/NRE-141198, PMID: 25547773

[76] Yu EH Min JH Shin YI Ko HY Ko SH. Effect of repetitive transcranial magnetic stimulation on post-stroke non-fluent aphasia in relation with Broca's area. Brain Neurorehabil. (2021) 14:e15. doi: 10.12786/bn.2021.14.e15, PMID: 36743437 PMC9879501

[77] Zheng K Chen M Shen Y Xu X Gao F Huang G . Cerebellar continuous Theta burst stimulation for aphasia rehabilitation: study protocol for a randomized controlled trial. Front Aging Neurosci. (2022) 14:909733. doi: 10.3389/fnagi.2022.909733, PMID: 35721014 PMC9201405

[78] Chen F Wang X Zhan C Yang L Wang Y Sun x . Therapeutical effect of low-frequency repetitive transcranial magnetic stimulation on cerebral infarction aphasia and its possible mechanism. Chin J Cerebrovasc Dis. (2012) 6:246–51. doi: 10.3877/cma.j.issn.1673-9248.2012.05.002

[79] Chen F Wang X Sun X Ke SH Wang Y Zhao X . Therapeutical effect of low-frequency repetitive transcranial magnetic stimulation on cerebral infarction aphasia and its effect on brain electrical activity. Chin J Cerebrovasc Dis. (2011) 5:96–101. doi: 10.3969/j.issn.1672-9248.2011.02.003

[80] Barwood CH Murdoch BE Whelan BM Lloyd D Riek S O’ Sullivan JD . Improved language performance subsequent to low-frequency rtms in patients with chronic non-fluent aphasia post-stroke. Eur J Neurol. (2011) 18:935–43. doi: 10.1111/j.1468-1331.2010.03284.x, PMID: 21138505

[81] Sang B Deng S Zhai J Hao T Zhuo B Qin C . Does acupuncture therapy improve language function of patients with aphasia following ischemic stroke? A systematic review and meta-analysis. NeuroRehabilitation. (2022) 51:231–45. doi: 10.3233/NRE-220007, PMID: 35527577 PMC9535561

[82] Harvey DY Podell J Turkeltaub PE Faseyitan O Coslett HB Hamilton RH. Functional reorganization of right prefrontal cortex underlies sustained naming improvements in chronic aphasia via repetitive transcranial magnetic stimulation. Cogn Behav Neurol. (2017) 30:133–44. doi: 10.1097/WNN.0000000000000141, PMID: 29256908 PMC5797702

[83] Zumbansen A Black SE Chen JL J Edwards D Hartmann A Heiss WD . Non-invasive brain stimulation as add-on therapy for subacute post-stroke aphasia: a randomized trial (Northstar). Eur Stroke J. (2020) 5:402–13. doi: 10.1177/2396987320934935, PMID: 33598559 PMC7856587

[84] Thiel A Black SE Rochon EA Lanthier S Hartmann A Chen JL . Non-invasive repeated therapeutic stimulation for aphasia recovery: a multilingual, multicenter aphasia trial. J Stroke Cerebrovasc Dis. (2015) 24:751–8. doi: 10.1016/j.jstrokecerebrovasdis.2014.10.021, PMID: 25735707

[85] Rubi-Fessen I Hartmann A Huber W Fimm B Rommel T Thiel A . Add-on effects of repetitive transcranial magnetic stimulation on subacute aphasia therapy: enhanced improvement of functional communication and basic linguistic skills. A randomized controlled study. Arch Phys Med Rehabil. (2015) 96:1935–1944.e2. doi: 10.1016/j.apmr.2015.06.017, PMID: 26189201

[86] Winhuisen L Thiel A Schumacher B Kessler J Rudolf J Haupt WF . Role of the contralateral inferior frontal gyrus in recovery of language function in poststroke aphasia: a combined repetitive transcranial magnetic stimulation and positron emission tomography study. Stroke. (2005) 36:1759–63. doi: 10.1161/01.STR.0000174487.81126.ef, PMID: 16020770

[87] Weiduschat N Thiel A Rubi-Fessen I Hartmann A Kessler J Merl P . Effects of repetitive transcranial magnetic stimulation in aphasic stroke: a randomized controlled pilot study. Stroke. (2011) 42:409–15. doi: 10.1161/STROKEAHA.110.597864 21164121

[88] Martin PI Treglia E Naeser MA Ho MD Baker EH Martin EG . Language improvements after TMS plus modified CILT: pilot, open-protocol study with two, chronic nonfluent aphasia cases. Restor Neurol Neurosci. (2014) 32:483–505. doi: 10.3233/RNN-130365, PMID: 25015701 PMC4592134

[89] Garcia G Norise C Faseyitan O Naeser MA Hamilton RH. Utilizing repetitive transcranial magnetic stimulation to improve language function in stroke patients with chronic non-fluent aphasia. J Vis Exp. (2013) 77:e50228. doi: 10.3791/50228-v PMC3731176 23852365

[90] Naeser MA Martin PI Theoret H Kobayashi M Fregni F Nicholas M . Tms suppression of right pars triangularis, but not pars opercularis, improves naming in aphasia. Brain Lang. (2011) 119:206–13. doi: 10.1016/j.bandl.2011.07.005, PMID: 21864891 PMC3195843

[91] Naeser MA Martin PI Lundgren K Klein R Kaplan J Treglia E . Improved language in a chronic nonfluent aphasia patient after treatment with CPAP and TMS. Cogn Behav Neurol. (2010) 23:29–38. doi: 10.1097/WNN.0b013e3181bf2d20, PMID: 20299861 PMC2939495

[92] Hamilton RH Sanders L Benson J Faseyitan O Norise C Naeser M . Stimulating conversation: enhancement of elicited propositional speech in a patient with chronic non-fluent aphasia following transcranial magnetic stimulation. Brain Lang. (2010) 113:45–50. doi: 10.1016/j.bandl.2010.01.001, PMID: 20159655 PMC2909623

[93] Naeser MA Martin PI Nicholas M Baker EH Seekins H Helm-Estabrooks N . Improved naming after TMS treatments in a chronic, global aphasia patient – case report. Neurocase. (2005) 11:182–93. doi: 10.1080/13554790590944663, PMID: 16006338 PMC1307171

[94] Ding X Zhang S Huang W Zhang S Zhang L Hu J . Comparative efficacy of non-invasive brain stimulation for post-stroke aphasia: a network meta-analysis and meta-regression of moderators. Neurosci Biobehav Rev. (2022) 140:104804. doi: 10.1016/j.neubiorev.2022.104804 35926728

[95] Grönberg A Henriksson I Stenman M Lindgren AG. Incidence of aphasia in ischemic stroke. Neuroepidemiology. (2022) 56:174–82. doi: 10.1159/000524206, PMID: 35320798

[96] Bucur M Papagno C. Are transcranial brain stimulation effects long-lasting in post-stroke aphasia? A comparative systematic review and meta-analysis on naming performance. Neurosci Biobehav Rev. (2019) 102:264–89. doi: 10.1016/j.neubiorev.2019.04.019, PMID: 31077693

[97] Johnson L Yourganov G Basilakos A Newman-Norlund RD Thors H Keator L . Functional connectivity and speech entrainment speech entrainment improves connectivity between anterior and posterior cortical speech areas in non-fluent aphasia. Neurorehabil Neural Repair. (2022) 36:164–74. doi: 10.1177/15459683211064264, PMID: 34968159 PMC8982955

[98] Chang WK Park J Lee JY Cho S Lee J Kim WS . Functional network changes after high-frequency RTMS over the Most activated speech-related area combined with speech therapy in chronic stroke with non-fluent aphasia. Front Neurol. (2022) 13:690048. doi: 10.3389/fneur.2022.690048, PMID: 35222235 PMC8866644

[99] Vuksanović J Jelić MB Milanović SD Kačar K Konstantinović L Filipović SR. Improvement of language functions in a chronic non-fluent post-stroke aphasic patient following bilateral sequential theta burst magnetic stimulation. Neurocase. (2015) 21:244–50. doi: 10.1080/13554794.2014.890731 24579976

[100] Iorga M Higgins J Caplan D Zinbarg R Kiran S Thompson CK . Predicting language recovery in post-stroke aphasia using behavior and functional MRI. Sci Rep. (2021) 11:8419. doi: 10.1038/s41598-021-88022-z, PMID: 33875733 PMC8055660

[101] Li H Zhang H Xu S Wang M Zhang J Liu J . Altered spontaneous brain activity in Poststroke aphasia: a resting-state fmri study. Brain Sci. (2023) 13:300. doi: 10.3390/brainsci13020300 36831843 PMC9954170

[102] Carson RG . Inter-hemispheric inhibition sculpts the output of neural circuits by co-opting the two cerebral hemispheres. J Physiol. (2020) 598:4781–802. doi: 10.1113/JP279793, PMID: 32770748

[103] Cai W Mueller C Li YJ Shen WD Stewart R. Post stroke depression and risk of stroke recurrence and mortality: a systematic review and meta-analysis. Ageing Res Rev. (2019) 50:102–9. doi: 10.1016/j.arr.2019.01.013, PMID: 30711712

