# Supplementary material for: Repetitive transcranial magnetic stimulation for post-stroke non-fluent aphasia: a systematic review and meta-analysis of randomized controlled trials
Source: Front Neurol. 2024 May 1;15:1348695. doi: 10.3389/fneur.2024.1348695 (PMC11094331; doi:10.3389/fneur.2024.1348695)
Supplement: Supplementary file 1 [file Data_Sheet_1.docx]

**Table. 1** Egger test for each outcome

| Outcome | Egger's test (P value) |
| --- | --- |
| Naming | 0.099 |
| Spontaneous Speech | 0.095 |
| Repetition | 0.273 |
| Aphasia quotient | 0.719 |

**Fig. 1** Process of literature screening

Studies included in

qualitative analysis (n=59)

Studies included in

meta-analysis (n=47)

Dentification

Screening

Eligibility

y

Included

d

Records after database

search (n=1244)

Screened titles and abstracts (n=607)

(n=1889)

Duplicate records (n=637)

Unrelated records (n=465)

Not RCTs (n=5)

Not non-fluent aphasia (n=21)

Not rTMS (n=12)

Data duplication (n=6)

No relevant outcome indicators (n=39)

Outcome indexes could not be merged (n=5)

Different treatment patterns of rTMS (n=7)

Full-text papers assessed for eligibility (n=142)

**Fig.2A** Summary graph of author judgments for each risk of bias criteria.

**Fig. 2B** Risk of bias assessment based on author judgment for individual studies.
